# Development of a generic β-lactamase screening system for improved signal peptides for periplasmic targeting of recombinant proteins in *Escherichia coli*

**DOI:** 10.1038/s41598-018-25192-3

**Published:** 2018-05-03

**Authors:** Tania Selas Castiñeiras, Steven G. Williams, Antony Hitchcock, Jeffrey A. Cole, Daniel C. Smith, Tim W. Overton

**Affiliations:** 1 Cobra Biologics, Stephenson Building, The Science Park, Keele, ST5 5SP UK; 2 School of Chemical Engineering, The University of Birmingham, Edgbaston, Birmingham, B15 2TT UK; 3 Institute of Microbiology & Infection, The University of Birmingham, Edgbaston, Birmingham, B15 2TT UK; 4 School of Biosciences, The University of Birmingham, Edgbaston, Birmingham, B15 2TT UK

## Abstract

Targeting of recombinant proteins to the *Escherichia coli* periplasm is a desirable industrial processing tool to allow formation of disulphide bonds, aid folding and simplify recovery. Proteins are targeted across the inner membrane to the periplasm by an N-terminal signal peptide. The sequence of the signal peptide determines its functionality, but there is no method to predict signal peptide function for specific recombinant proteins, so multiple signal peptides must be screened for their ability to translocate each recombinant protein, limiting throughput. We present a screening system for optimising signal peptides for translocation of a single chain variable (scFv) antibody fragment employing TEM1 β-lactamase (Bla) as a C-terminal reporter of periplasmic localisation. The *Pectobacterium carotovorum* PelB signal peptide was selected as the starting point for a mutagenic screen. β-lactamase was fused to the C-terminal of scFv and β-lactamase activity was correlated against scFv translocation. Signal peptide libraries were generated and screened for β-lactamase activity, which correlated well to scFv::Bla production, although only some high activity clones had improved periplasmic translocation of scFv::Bla. Selected signal peptides were investigated in fed-batch fermentations for production and translocation of scFv::Bla and scFv without the Bla fusion. Improved signal peptides increased periplasmic scFv activity by ~40%.

## Introduction

The Gram negative bacterium *Escherichia coli* is a mainstay of the biopharmaceutical industry, and is the most common non-mammalian cell production system for recombinant protein biopharmaceuticals^1^. Advantages of *E*. *coli* include a long history of safe use, high biomass and protein yields, and ease of genetic engineering^2^. *E*. *coli* is used for the production of relatively simple recombinant protein biopharmaceuticals such as insulin, Growth Hormone and Granulocyte-Colony Stimulating Factor^1^. Whereas *E*. *coli* lacks the ability to make many post-translational modifications (such as glycosylation) that are required for some recombinant protein biopharmaceuticals, which necessitate the use of eukaryotic hosts such as CHO cells, it is able to generate disulphide bonds between cysteine residues. Disulphide bonding in *E*. *coli* naturally occurs in the periplasm, catalysed by the Dsb proteins (reviewed by^3^). Therefore, recombinant proteins must be transported into the periplasm in order for disulphide bonding to occur. This is of particular relevance to antibody fragments which often require disulphide bonding for correct folding and function^4^. An example is the human biopharmaceutical Certolizumab pegol (Cimzia^®^), which is a PEGylated anti-Tumour Necrosis Factor α antigen-binding (Fab) antibody fragment produced in *E*. *coli*^5^. In addition to disulphide bonding, periplasmic targeting can decrease proteolysis of recombinant proteins by cytoplasmic proteases, and allows simplified product release and capture without the need for whole cell lysis^6^.

*E*. *coli* exploits multiple mechanisms for transport of proteins into the periplasm that include the SecB, SRP and twin-arginine (Tat) pathways (reviewed by^7,8^). The SecB and SRP pathways both employ a common transport mechanism. The SecYEG complex comprises a pore in the inner membrane, which transports unfolded polypeptide chains from the cytoplasm to the periplasm. The SecB pathway is post-translational, whereby polypeptide chains are translocated after complete translation, whereas the SRP pathway is co-translational, as translocation occurs while the polypeptide chain is still being translated by the ribosome. The third mechanism, Tat, consists of a larger pore made up of the TatABC proteins, which is able to transport fully folded proteins into the periplasm. Although the Tat system has recently been successfully developed for recombinant protein production (RPP) applications^9^, the majority of recombinant proteins translocated to the periplasm have been directed via the SecB and SRP pathways.

Targeting of polypeptide chains to the periplasm via SecB, SRP or Tat requires an N-terminal signal peptide that specifically interacts with components of the three pathways. This signal peptide is cleaved from the polypeptide chain by a protease during translocation, resulting in a mature protein in the periplasm. The destination (cytoplasmic or periplasmic) and route (SecB, SRP or Tat) of the polypeptide chain is therefore specified by the sequence of the signal peptide. Multiple factors affect the functionality of the signal peptide. It must interact, via electrostatic and hydrophobic interactions, with the inner membrane and the translocation apparatus to facilitate polypeptide transport^10^. The increased incidence of rare codons in the signal peptide has been revealed to play a role in control of translation speed and protein folding (reviewed by^11^). The structure of the mRNA encoding the signal peptide has also been shown to have an influence on translocation in *Lactococcus lactis*^12^, and in *E*. *coli* via translational pausing^13^. Therefore, the signal peptide affects protein translation and translocation via a variety of mechanisms.

Selection and optimisation of signal peptides for effective translocation of recombinant proteins to the periplasm has been the subject of much research (reviewed by^14^). There are several important factors to consider when selecting a signal peptide and matching it to a RPP process. First, the rate of recombinant protein translation must match the rate of transport to the periplasm to ensure that unfolded polypeptides do not accumulate in the cytoplasm^15^. This would increase the risk of protein misfolding and inclusion body formation, thus inducing the cytoplasmic heat shock response^16^. Second, recombinant protein translocation must not prevent translocation of native proteins, which would negatively affect bacterial physiology and viability. These requirements for the design of periplasmic RPP are additional to the requirements for any RPP process, such as balancing the metabolic requirements of RPP and growth to prevent metabolic burden or the stringent response, and prevention of excessive protein misfolding that would trigger the heat shock response (reviewed by^2^). Therefore signal peptide choice is one element of process design, along with optimisation of fermentation conditions^17^.

This study stemmed from a simple question: “Which signal peptide should be used for targeting a recombinant protein to the periplasm?” The answer to this question is far from straightforward and cannot at this time be answered generically. There is no way at present to select or design a signal peptide *in silico* based on the nucleotide or peptide sequence of the recombinant protein (RP) of interest. Both academic and industrial groups have approached the problem by using multiple signal peptides and analysing RPP and periplasmic export; a “trial and error” approach (reviewed by^14^). Bioinformatic analysis has been used to construct the consensus signal peptide for *E*. *coli*^18^; however, this was found to be suboptimal for periplasmic production of a single chain variable (scFv) antibody fragment, reinforcing the need for experimental selection of optimal signal peptides for each recombinant protein^19^. Mutagenic screening approaches have sought to identify optimal signal peptides in *E*. *coli*^20^, *Bacillus subtilis*^21^ and *Lactococcus lactis*^12^. However, for *E*. *coli*, selection of optimal signal peptides for one recombinant protein does not translate to a generic signal peptide that is suitable for other recombinant proteins, reinforcing the need for rapid screening procedures^20^. Unfortunately, analysis of periplasmic targeting of RPs is time-consuming and low throughput without extensive robotics, requiring separation of cytoplasmic and periplasmic protein fractions followed by SDS-PAGE.

The primary aim of the current work was to develop a method for screening randomly produced libraries for signal peptides that improve the periplasmic targeting of any specific protein of interest. Our approach combined several well-established rapid techniques to generate and detect colonies that merit more detailed analysis. They include the use of error-prone PCR for library generation, C-terminal β-lactamase fusion to report secretion of the target into the periplasm^22^ and nitrocefin to detect β-lactamase activity of colonies after overnight growth^23^. We started with a model scFv antibody fragment, 13R4^24^, targeted to the periplasm using three commonly-used signal peptides. Detection of periplasmic scFv was then simplified and accelerated using a C-terminal TEM-1 β-lactamase (Bla) fusion. Signal peptide libraries were generated using error-prone PCR and chemical oligonucleotide synthesis routes and independently cloned upstream of the scFv::*bla* fusion to generate libraries, which were screened using β-lactamase assays. This assay system allowed higher-throughput screening using microplates, which would be difficult to achieve for the subcellular fractionation of *E*. *coli* and analysis of proteins. Promising clones were then grown in small scale and bioreactor cultures and scFv production and location assessed. Finally, the system was validated using selected signal peptide-scFv constructs minus their *bla* fusion.

## Results and Discussion

### Production of the scFv 13R4 using existing signal sequences

**Figure 1:**
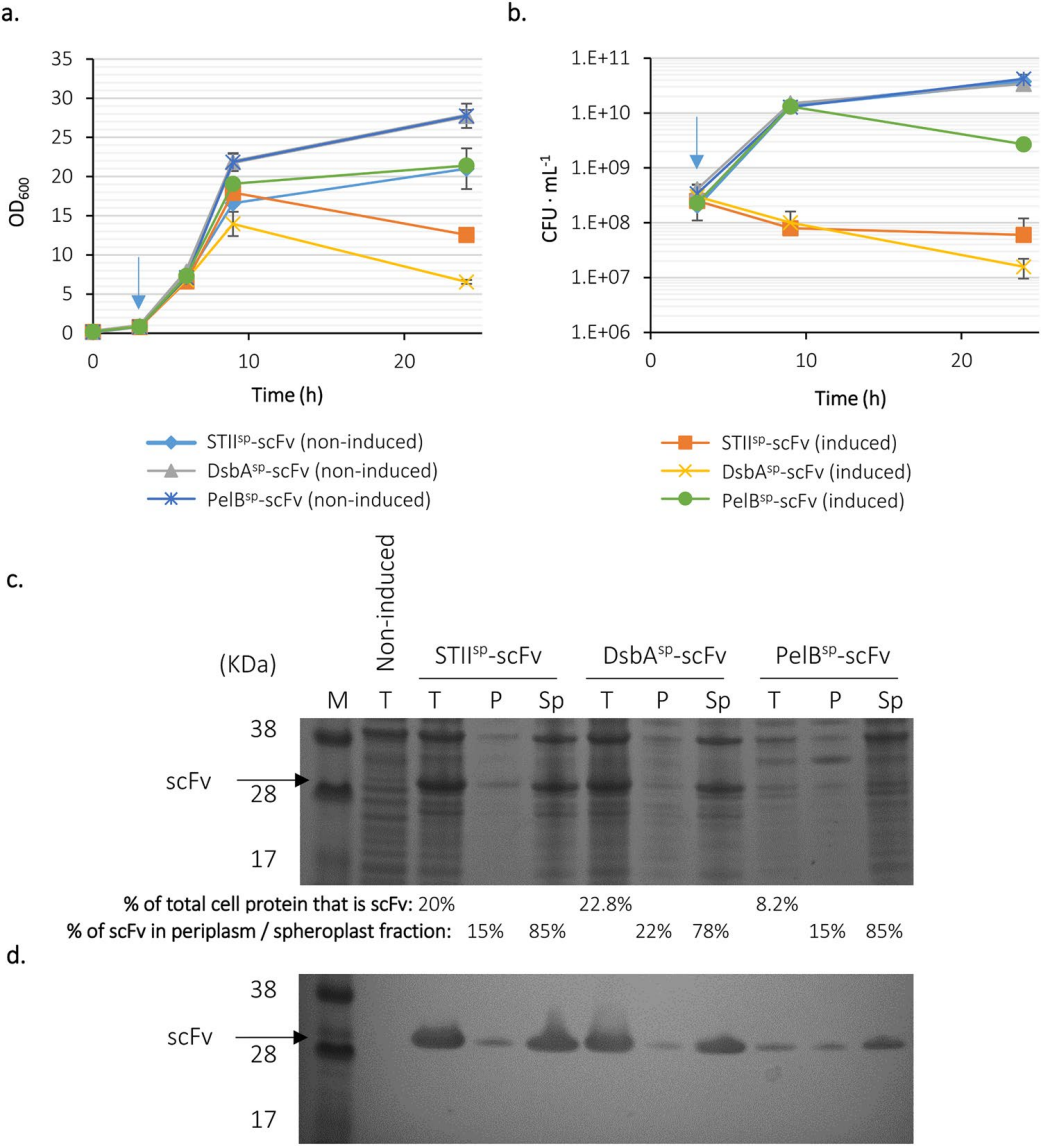
Production of scFv 13R4 in the periplasm using STII, DsbA and PelB signal peptides. *E*. *coli* BL21-A carrying the vector encoding scFv 13R4 fused to STII^sp^, DsbA^sp^ or PelB^sp^ was grown at 25 °C; RPP was induced with 0.02% arabinose at an OD_600_ ≈ 0.5 (blue arrow). Upon induction, 0.25% glucose was added to the culture medium. (**a**) The OD_600_ and (**b**) CFU of the culture were measured over time. Data are shown as mean values from replica flasks, error bars are ±1 standard deviation. (**c**) SDS-PAGE and (**d**) Western blot analysis (anti-His) of total (T), periplasm (P) and spheroplast (Sp) fractions after 24 hours growth. A sample of the culture carrying STII^sp^ fused to scFv 13R4 grown under non-induced conditions was used as control. Percentages of total cell protein that was scFv and the distribution of scFv between periplasmic and spheroplast fractions shown below (**c**). Uncropped gel images are available in Supplemental Fig. S12.

Initial experiments sought to produce the model scFv 13R4 (referred to henceforth as scFv; 24), in the periplasm. As with previous studies, a variety of signal peptides were used: the STII signal peptide (STII^sp^), which targets *E*. *coli* heat stable enterotoxin II via SecB^25^; PelB^sp^, from *Pectobacterium carotovorum* (also SecB-dependent^26^) and DsbA^sp^, which targets *E*. *coli* disulphide oxidoreductase A via the SRP pathway^27^. Each signal peptide was cloned upstream of the gene encoding scFv 13R4 on plasmid pLBAD2; expression was driven by the arabinose-inducible pBAD promoter^28^. Transformed *E*. *coli* BL21-A cultures were grown in terrific broth (TB) at 25 °C and RPP was induced at an OD_600_ of 0.5 with 0.02% (w/v) arabinose to activate the pBAD promoter and 0.25% (w/v) glucose to regulate RPP by catabolite repression, conditions previously optimised for production of this scFv. Growth data revealed that induction of RPP inhibited growth of STII^sp^-scFv and DsbA^sp^-scFv cultures (Fig. 1a); CFU analysis corroborated this by way of decreased culturability, indicating stress (Fig. 1b), whereas PelB^sp^-scFv cultures displayed less growth inhibition and a lower decrease in culturability. SDS-PAGE analysis of bacteria harvested after 24 hours growth revealed higher accumulation of scFv in STII^sp^-scFv and DsbA^sp^–scFv than PelB^sp^-scFv cultures, although relatively little transport to the periplasm and virtually none in the case of DsbA^sp^ (Fig. 1c,d). In summary: STII^sp^ gave rise to relatively high scFv production (20% of total cell protein, TCP), moderate periplasmic accumulation (only 15% of scFv was in the periplasmic fraction) but poor growth; DsbA^sp^ high scFv production (22.8% of TCP), high periplasmic accumulation (22% of TCP) and poor growth; and PelB^sp^ moderate scFv production (8.2% of TCP), moderate periplasmic accumulation (15%) and better growth. These data confirm that choice of signal sequence has a large impact on not only translocation but also overall production of scFv 13R4, and significant effects on bacterial growth.

### Development of a more high-throughput method for assessing periplasmic targeting

Since growth followed by harvest and separation of bacteria into periplasmic and spheroplast fractions and analysis by SDS-PAGE is a time-consuming process, and unsuited to use as a high throughput screening method without the involvement of robotic platforms, we utilised the TEM-1 β-lactamase (Bla) as a reporter of periplasmic location^22^. Bla confers resistance to β-lactam antibiotics such as penicillin by cleaving the β-lactam ring. The Bla protein is targeted to the periplasm, the site of action of β-lactam antibiotics. Mature, periplasmic Bla also contains a single disulphide bond, although disulphide bonding is not required for activity^29^. Bla has previously been used as a C-terminal fusion reporter of periplasmic recombinant protein folding^22^ and as a screening tool for selection of improved *E*. *coli* signal peptides^20^, although in the latter study not as a fusion to recombinant proteins.

**Figure 2:**
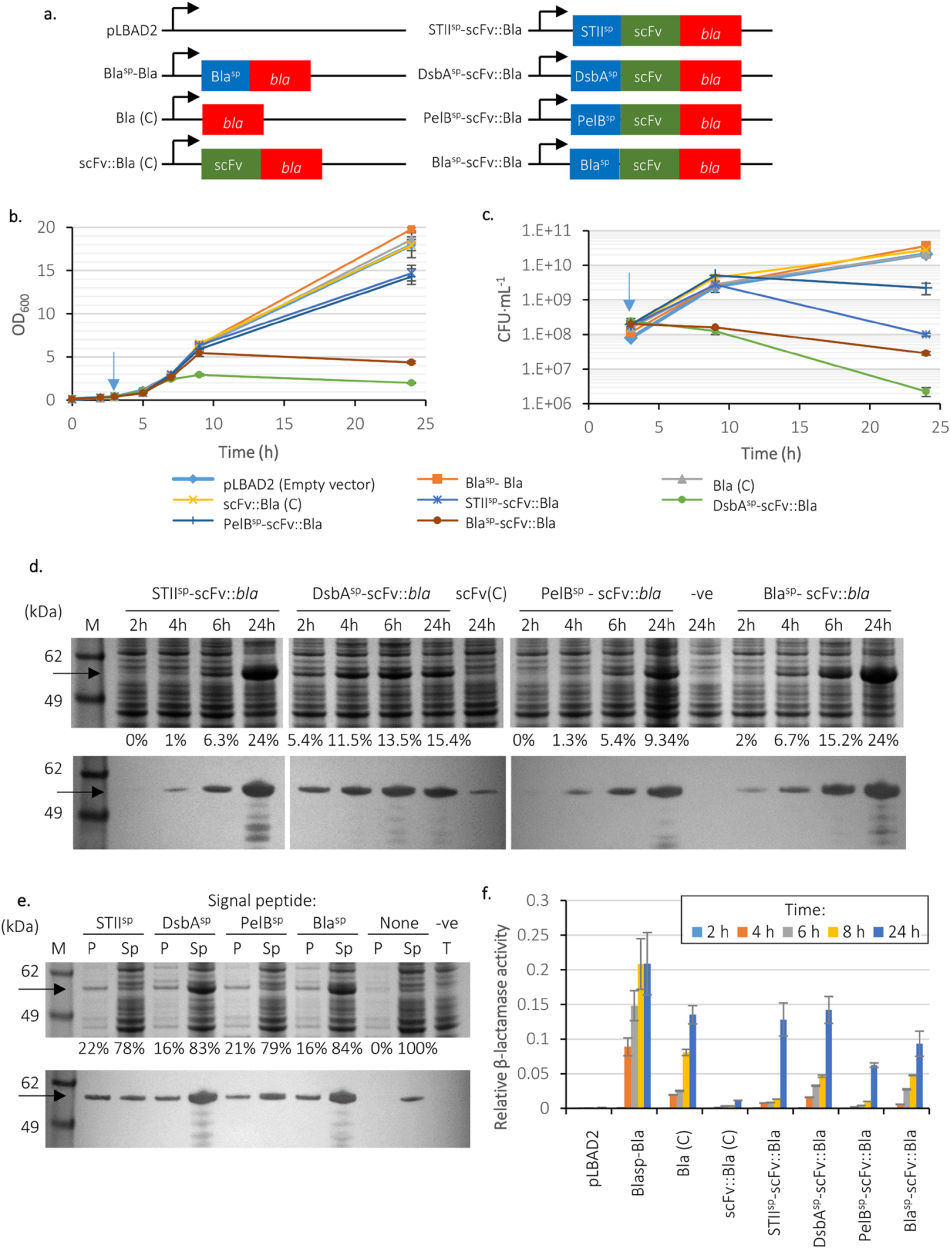
Construction and characteristics of signal peptide-scFv-β-lactamase constructs. (**a**) Plasmid constructs generated for the β-lactamase screening; signal peptide (blue), scFv 13R4 (green) and the *bla* reporter (red). Cytoplasmic versions of β-lactamase and scFv::Bla without a signal peptide are included as controls, marked as (C). *E*. *coli* BL21-A carrying the vectors in (**a**) were grown at 25 °C, induced with 0.02% of arabinose at an OD_600_ ≈ 0.5 (blue arrow), and the OD_600_ **(b)** and CFU **(c)** measured. Data are shown as mean values from replica flasks, error bars are ±1 standard deviation. **(d)** Cells were harvested and analysed by SDS-PAGE and Western blotting (anti myc). The quantity of scFv::Bla is expressed as a percentage of whole cell protein at the bottom of the gel. Controls are scFv::Bla (C) and empty vector (−ve). Percentages of total cell protein that was scFv are shown below the gel. **(e)** The periplasmic (P) and spheroplast (Sp) fractions of samples taken after 6 hours growth were separated and analysed by SDS-PAGE and Western blotting using anti-myc. Negative control is the empty vector (pLBAD2), total protein. Percentage distributions of scFv between periplasmic and spheroplast fractions are shown below the gel. **(f)** β-lactamase activity was determined using nitrocefin. Transformants were grown at 25 °C and induced with 0.02% arabinose after 2 hours growth; samples were taken at intervals and β-lactamase activity of whole cells was measured. Error bars represent the 5% uncertainty in the calculation of the slopes corresponding to β-lactamase activity (n = 3), which is expressed in terms of change in OD_495_ per minute per OD_600_. Uncropped gel images are available in Supplemental Fig. S13.

The gene encoding scFv 13R4 was fused at its C-terminus to the *bla* gene from pUC18; four different signal peptide sequences were used (STII^sp^, DsbA^sp^, PelB^sp^ and the native Bla^sp^; Fig. 2a). In addition, controls comprising scFv-*bla* without a signal sequence and *bla* with and without its native signal sequence were constructed. Resultant constructs were transformed into *E*. *coli* BL21-A and transformants were grown at 25 °C with induction (0.02% arabinose) at OD_600_ = 0.5. Growth analysis (Fig. 2b,c) revealed repression of growth and/or culturability in all cultures expressing periplasmic scFv (in order of decreasing severity) DsbA^sp^-scFv::*bla*, >Bla^sp^-scFv::*bla* >STII^sp^-scFv::*bla* >PelB^sp^-scFv::*bla*. These data confirm growth repression observed in cultures expressing scFv without the *bla* fusion (Fig. 1).

SDS-PAGE and Western blot analysis of cultures expressing *bla* with and without the Bla signal peptide confirmed expression and the requirement of Bla^sp^ to direct Bla to the periplasm (Supplemental Fig. S1). Bla was also observed in the medium of cultures expressing periplasmic Bla, indicating some leakage. SDS-PAGE and Western blot analysis of cultures expressing scFv::*bla* revealed relatively high accumulation of scFv::Bla in STII^sp^ and Bla^sp^ cultures (both having 24% of TCP as scFv::Bla at 24 h), lower accumulation with DsbA^sp^ (15%), and lowest accumulation with PelB^sp^ (9%; Fig. 2d). The proportion of scFv::Bla in the periplasm was highest with STII^sp^(22%), followed by PelB^sp^ (21%), then DsbA^sp^ and Bla^sp^ (both 16%; Fig. 2e).

To simplify quantification of periplasmic transport of scFv::Bla fusions, the activity of β-lactamase was quantified using two methods. First, the minimal inhibitory concentration (MIC) of the β-lactam antibiotic ampicillin was determined for cultures expressing scFv::*bla* fusions. Transformants were grown in liquid culture without induction of RPP, then serially diluted and plated onto M-H agar plates supplemented with kanamycin, 0.2% or 0.02% arabinose (to induce RPP) and concentrations of ampicillin from 3 to 1600 µg·mL^−1^. Plates containing 0.2% arabinose were incubated at 37 °C and plates containing 0.02% arabinose at 25 °C; colonies were enumerated (Table 1; CFU data presented in Supplemental Fig. S2). A signal peptide was required to confer resistance to ampicillin. For cultures grown at 37 °C and induced with 0.2% arabinose, the DsbA^sp^ and Bla^sp^ did not confer ampicillin resistance. As these two signal peptides were deleterious to growth and culturability even during culture at 25 °C (low-stress conditions; Fig. 2b,c), it was concluded that this was most likely due to a lack of viability at 37 °C in the presence of arabinose (high-stress conditions). Growth was detected on the control plates (containing kanamycin but not induced with arabinose) for DsbA^sp^ and Bla^sp^, confirming this hypothesis. Growth at 25 °C and induction with 0.02% arabinose restored ampicillin resistance for all periplasmically-targeted scFv::*bla* fusions. Ampicillin agar plates could thereby not only select for clones generating high quantities of scFv::Bla, but also counterselect against clones with growth defects due to RPP.

**Table 1:** Minimum inhibitory concentrations for ampicillin of cultures expressing scFv::*bla* with different signal peptides.

	MIC at 37 °C (mg·L^−1^)	MIC at 25 °C (mg·L^−1^)
Empty vector	3	3
Bla^sp^-*bla*	>1600	400
*bla*	3	3
scFv::*bla*	3	3
STII^sp^-scFv::*bla*	400	200
DsbA^sp^-scFv::*bla*	NG	800
PelB^sp^-scFv::*bla*	800	100
Bla^sp^-scFv::*bla*	NG	200

NG: No growth on any plate containing arabinose to induce production of recombinant protein.

Second, β-lactamase activities were determined using the chromogenic β-lactam nitrocefin as a substrate^30^. Cells were grown in liquid culture at 25 °C and RPP was induced with 0.02% arabinose, after which β-lactamase activity of whole cells was measured at intervals (Fig. 2f). As reported previously, cytoplasmically targeted Bla was active due to the lack of a requirement of disulphide bonding for enzymatic activity^29,31^. However, the β-lactamase activity of cytoplasmic scFv::Bla was very low, presumably due to relatively low accumulation of scFv::Bla in the cytoplasm, possibly due to protease degradation (Fig. 2d, lane scFv(C)). All four signal peptides permitted some level of activity from scFv::Bla. DsbA^sp^ and Bla^sp^ displayed relatively high activity after 4 hours, reflecting the rapid accumulation of scFv::Bla observed in SDS-PAGE (Fig. 2d). PelB^sp^ had both the lowest scFv::Bla accumulation as judged by SDS-PAGE and β-lactamase activity. Overall, we have demonstrated that β-lactamase fusions are a good method for evaluation of periplasmic targeting of scFv 13R4.

### Development of a screen for selecting improved signal peptides

**Figure 3:**
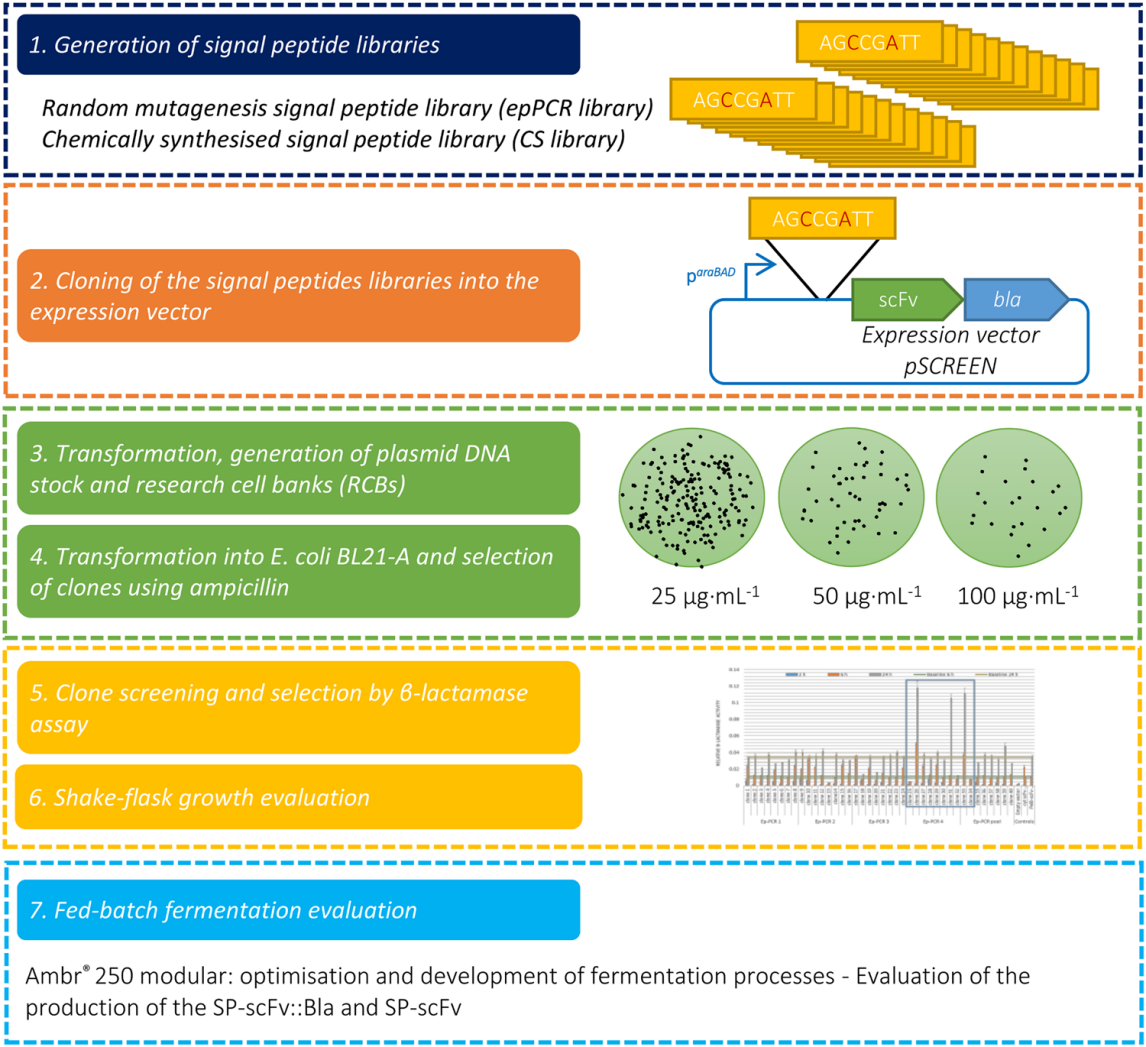
Workflow for β-lactamase screening for periplasmic protein production.

Based on the successful testing of scFv::Bla fusions, a screen was developed to select optimal signal peptides. The design of the screen is summarised in Fig. 3. Two random libraries of signal peptides were generated, one using error-prone PCR (epPCR) and one chemically synthesised (CS). The libraries were cloned upstream of scFv::*bla* fusions in the plasmid pSCREEN (construction detailed in Supplementary materials and methods) and the resulting colonies screened first for growth on agar plates containing ampicillin up to a concentration of of 200 μg·mL^−1^ (in effect an enrichment step), followed by β-lactamase assay using nitrocefin. Signal peptides were then selected on the basis of β-lactamase activity and analysed for growth and scFv::Bla production, first in shake flasks and then in fed-batch fermentation using an Ambr^®^ 250 modular fermentation system. Finally, fed-batch fermentation was repeated for the selected signal peptides directing translocation of the scFv without the Bla fusion. The PelB signal peptide was used as a starting point, as it allowed translocation of scFv and scFv::Bla to the periplasm but did not affect growth to a great extent in the above experiments.

### Error-prone PCR library

For the epPCR library, four successive epPCR reactions were used to introduce mutations into the PelB signal peptide. This approach was taken as the chosen enzyme (Genemorph II, Agilent) can introduce 9–16 mutations per kilobase. As the signal peptide sequence is only 66 nucleotides in length, four successive rounds of epPCR were used to introduce additional mutations, aiming for up to 8 nucleotide mutations per signal peptide. The resultant signal peptide pools were named epPCR1, epPCR2, epPCR3 and epPCR4, with a further pool (epPCR5) comprising a mixture of pools 1–4. The linearised screen plasmid (pSCREEN; Supplemental Fig. S3) was ligated with each signal peptide pool, electroporated into *E*. *coli* ElectroSHOX, recovered in LB medium without selection for one hour, then transferred to a flask and grown for 12–18 hours with selection, after which plasmid DNA was isolated and purified. The resultant plasmid library was then transformed into the expression strain BL21-A, serially diluted and plated onto agar plates containing: 0.2% arabinose to induce scFv::Bla production, kanamycin to select for transformants, and ampicillin at concentrations between 3 and 1600 µg·mL^−1^ to select for Bla^+^ bacteria. Plates were incubated at 37 °C. These growth conditions represent high induction so will counterselect for clones with low viability or those whose growth and physiology are negatively affected by RPP (Supplemental Fig. S2).

Growth was observed on agar plates at up to 200 µg·mL^−1^ ampicillin. Two hundred clones were selected at random (40 from each epPCR pool 1–5), and split into 5 libraries (A–E), that were grown in duplicate in 96-well plates in TB at 25 °C (conditions chosen as in Fig. 1 to permit effective production of scFv), and induced with 0.02% arabinose after 2 hours growth. Relative β-lactamase activities were determined for each clone (Supplemental Fig. S4) and compared to PelB^sp^-scFv::*bla*, scFv::*bla* without a signal peptide and the empty vector (pLBAD2) as controls. The majority of clones had a similar β-lactamase activity to PelB^sp^-scFv::*bla*. Some clones had a lower activity (eg epA22, denoting error-prone PCR library A clone 22, and epB3); some clones had higher activity than PelB^sp^-scFv::*bla* after 8 hours growth but no increase in activity after 24 hours (eg epA7, epA19). However, the higher activity of some clones after 24 hours growth showed not only that the current objective of high-level production of functional scFv::Bla after 8 hours growth had been achieved, but also suggests clone stability.

The robustness of the β-lactamase assay as a screening method was assessed by measurement of the β-lactamase activity of 40 different colonies of *E*. *coli* BL21-A transformed with the vector encoding PelB^sp^-scFv::Bla (Supplemental Fig. S5). Colony to colony variability was low after 8 hours growth (CV = 0.13), although slightly higher after 24 hours growth (CV = 0.17), likely reflecting heterologous bacterial responses to stress imposed by periplasmic scFv::Bla production. Nonetheless, colony to colony variability was far lower than clone to clone variability observed in the epPCR screen (Supplemental Fig. S4; CV = 0.58 at 8 h and 0.58 at 24 h), confirming the validity of this approach.

### Chemically synthesised signal peptide library screening

A similar workflow to the epPCR signal peptide library was followed for the chemically synthesised (CS) signal peptide library. The library was based on PelB^sp^ and comprised 10 000 signal peptides, designed to contain 2–4 mutations each. A further 200 clones were assayed for β-lactamase activity (Supplemental Fig. S6). Compared to the epPCR signal peptide library, there was a larger number of high activity clones in the CS library. This was not unexpected as the CS library was designed to contain a comparable number of mutations per signal peptide as the epPCR4 pool. As with the epPCR library, individual clones had higher, comparable or lower β-lactamase activity when compared to PelB^sp^. Some clones in the CS library had higher β-lactamase activity than the highest activity clones in the epPCR library (eg csA3, 0.142 units; csD4, 0.165 units; compared to epPCR library maximum epC28, 0.118 units).

### Evaluation of signal peptide clones from the epPCR library

**Figure 4:**
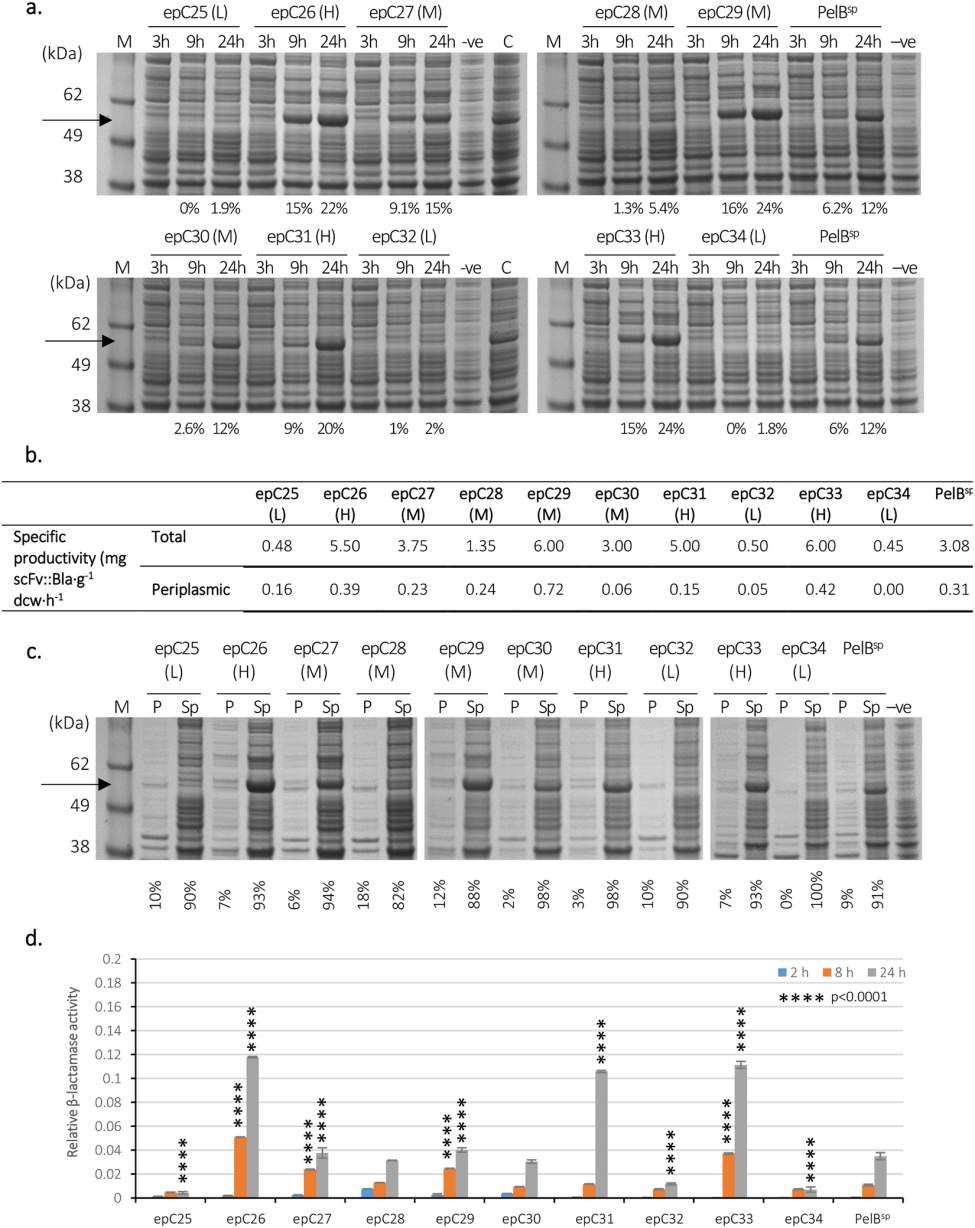
Production of scFv::Bla by clones selected from the epPCR library. *E*. *coli* BL21-A carrying vectors coding for different signal peptides from the epPCR library (epC25-epC34) or PelB^sp^ fused to scFv::Bla were grown at 25 °C and induced with 0.02% arabinose at an OD_600_ ≈ 0.5. (**a**) SDS-PAGE gels showing accumulation of scFv::Bla (black arrow) from whole cell lysates. Samples were obtained before (3 h) and after induction (9 and 24 hours post-inoculation). M is the molecular size marker. Cultures carrying PelB^sp^ fused to scFv::Bla, the cytoplasmic scFv::Bla (C) or the empty vector (-ve) after 24 hours were used as controls. (**b**) Specific productivity of total scFv and periplasmic scFv at 24 hours growth. Values calculated from densitometry data from panel (**a,c**) as described in supplemental materials and methods. (**c**) The periplasm (P) and spheroplast (Sp) fractions of samples after 9 hours of growth were analysed by SDS-PAGE. The quantity of the scFv::Bla is expressed as percentage of whole cell protein (**a**) and the percentage of protein accumulated in each fraction (**c**). (**d**) The β-lactamase activity of each clone evaluated here. Error bars represent the 5% uncertainty in the calculation of the slopes corresponding to β-lactamase activity (n = 2), which is expressed in terms of change in OD_495_ per minute per OD_600_. ****Indicates values statistically significant (P < 0.0001) compared to the control (PelB^sp^-scFv::Bla) after selecting adjusted p < 0.001 as the level of significance during statistical analysis of data. Uncropped gel images are available in Supplemental Fig. S14.

Ten clones were selected for further analysis to investigate the variability generated by the epPCR library, epC25-epC34, representing three phenotypic groups: high activity clones epC26, epC31 and epC33; clones with activity close to the wild type PelB^sp^ (epC27, epC28, epC29 and epC30); and clones with low activity (epC25, epC32 and epC34). Clones were grown in shake flasks in terrific broth (TB) at 25 °C and induced with 0.02% arabinose at OD_600_ = 0.5. Growth and CFU data comparing the 10 selected clones with PelB^sp^ (Supplemental Fig. S7) were inconclusive. Analysis of scFv accumulation by SDS-PAGE revealed that all high activity clones had higher scFv::Bla accumulation than the wild type PelB^sp^ (20–24% versus 12% of TCP was scFv::Bla at 24 h; Fig. 4a). Conversely, all low activity clones had lower scFv::Bla accumulation (around 2% of TCP); most ‘medium activity’ clones had comparable scFv::Bla accumulation to PelB^sp^. It should be noted that clone epC29 accumulates a large quantity of scFv (24% of TCP at 24 h), as revealed by SDS-PAGE, although has a ‘medium’ β-lactamase activity (~0.04 units at 24 h). This highlights that, although β-lactamase activity assays are a useful rapid screening technique to select for potential high-accumulating clones, SDS-PAGE must be used to measure protein concentration. Specific productivity values (Fig. 4b) confirm these observations.

However, fractionation of periplasmic and spheroplast fractions revealed that no high activity clones had an increased proportion of scFv::Bla in the periplasm compared to PelB^sp^ (Fig. 4c). Therefore, it can be concluded that the increase in activity in the high activity epPCR clones investigated here was primarily driven by an increase in overall scFv::Bla accumulation rather than an increased proportion of scFv::Bla being transported to the periplasm.

### Evaluation of signal peptide clones from the CS library

**Figure 5:**
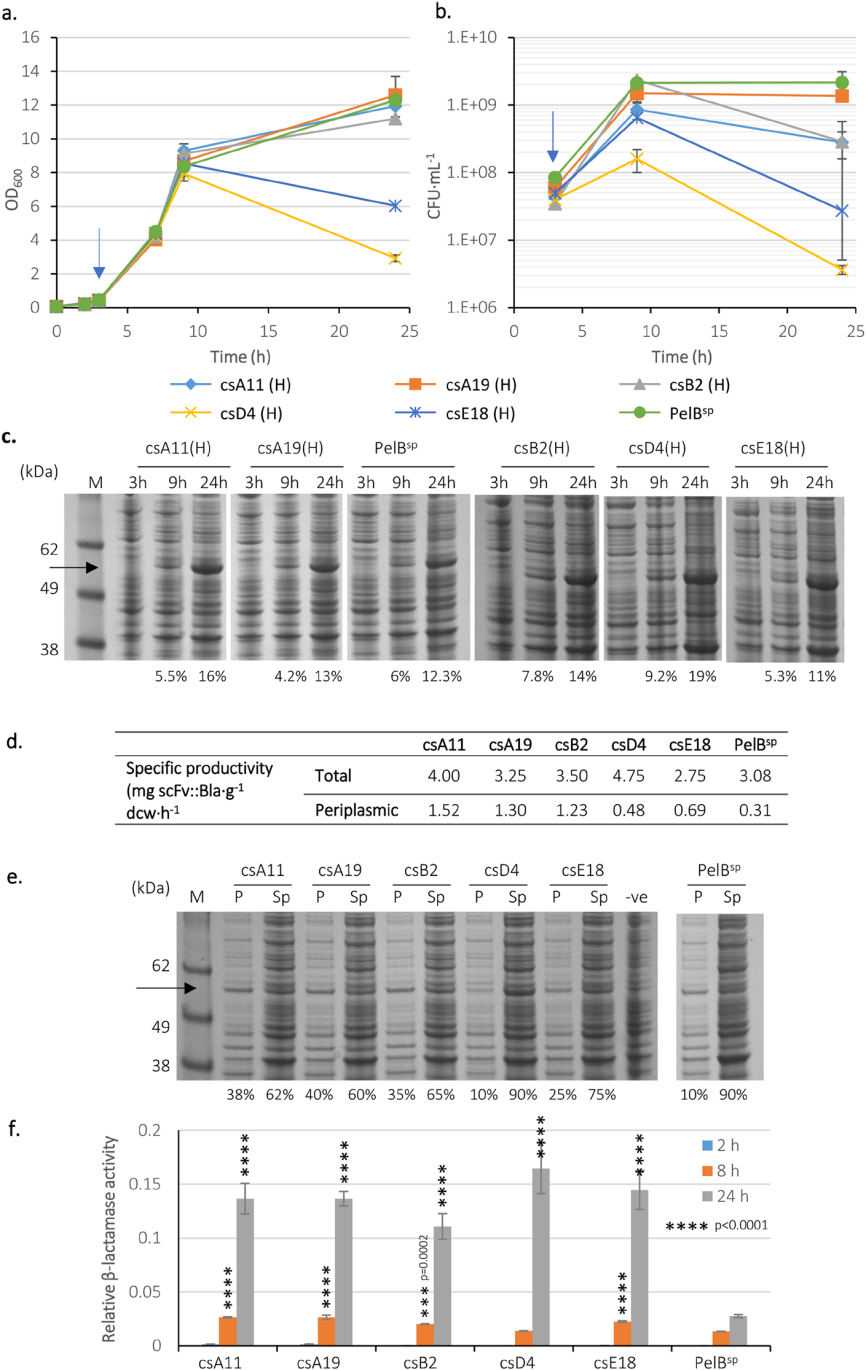
Analysis of selected clones from chemically synthesised (CS) signal peptide library. *E*. *coli* BL21-A carrying the vector encoding scFv::Bla with five high-activity signal peptides from the CS library were grown at 25 °C and induced with 0.02% arabinose at an OD_600_ ≈ 0.5 (blue arrow). The OD_600_ (**a**) and CFU (**b**) were measured. Data shown are mean values of two replica flasks and error bars are ±1 standard deviation (n = 2). (**c**) SDS-PAGE analysis of whole cell lysates obtained before (3 h) and after induction (9 and 24 hours post-inoculation). M is the molecular size marker. (**d**) Specific productivity of total scFv and periplasmic scFv at 24 hours growth. Values calculated from densitometry data from panels (**c**,**e**) as described in supplemental materials and methods. (**e**) Periplasm (P) and spheroplast (Sp) fractions from cultures after 9 hours of growth. Negative control is the empty vector (−ve) after 24 hours growth. The quantity of the scFv::Bla is expressed as percentage of whole cell protein (**c**) and the percentage of protein accumulated in each fraction (**e**). (**f**) The β-lactamase activity of each clone evaluated here. Error bars represent the 5% uncertainty in the calculation of the slopes corresponding to β-lactamase activity (n = 2), which is expressed in terms of change in OD_495_ per minute per OD_600_. Asterices indicate values statistically significant (****p < 0.0001; ***0.001 > p > 0.0001) compared to the control (PelB^sp^-scFv::Bla) after selecting adjusted p < 0.001 as the level of significance during statistical analysis of data. Uncropped gel images are available in Supplemental Fig. S15.

Five clones were selected from the CS library and analysed as above (Fig. 5). The β-lactamase activity of all five was higher than with PelB^sp^, and four had higher activity than the highest activity epPCR signal peptides. Growth data revealed that growth of clones csD4 and csE18 was inhibited following induction of RPP (Fig. 5a,b). SDS-PAGE analysis (Fig. 5c) revealed that none of the CS library clones analysed accumulated as much scFv::Bla as the high producers analysed from the epPCR screen (11–19% of TCP was scFv::Bla compared to 20–24% for the epPCR clones; Fig. 4a); in fact, two clones (csA19 and csE18) had comparable accumulation to the wild type PelB^sp^. Specific productivity data for total scFv confirmed this (Fig. 5d). However, analysis of periplasm and spheroplast fractions revealed increased proportions of scFv::Bla in the periplasm of four clones (25–40% of scFv being in the periplasmic fraction compared to 10% for PelB^sp^). In only one clone (csD4) was partitioning similar to PelB^sp^ (Fig. 5d). Specific productivity of periplasmic scFv was higher than PelB^sp^ for all four analysed clones, and much higher for the three best-growing clones (csA11, csA19 and csB2; 1.23 to 1.52 mg scFv·g^−1^ dcw·h^−1^ versus 0.31 for PelB^sp^).

Taken together, the data reveal two mechanisms by which elevated β-lactamase activity was achieved by clones from the mutant signal peptide libraries. First, overall accumulation of scFv::Bla was elevated (named here high expression signal peptides) as seen in all high activity epPCR library clones analysed and clone csD4 from the CS library. These signal peptides are likely to allow increased translation of the scFv::Bla but not enhance translocation to the periplasm. This phenomenon has been observed previously^32^. Second, increased partitioning of scFv::Bla into the periplasm was observed in CS library clones csA11, csA19, csB2 and csE18, suggesting enhanced periplasmic translocation (named here high transporting signal peptides).

### Sequence analysis of signal peptides

**Figure 6:**
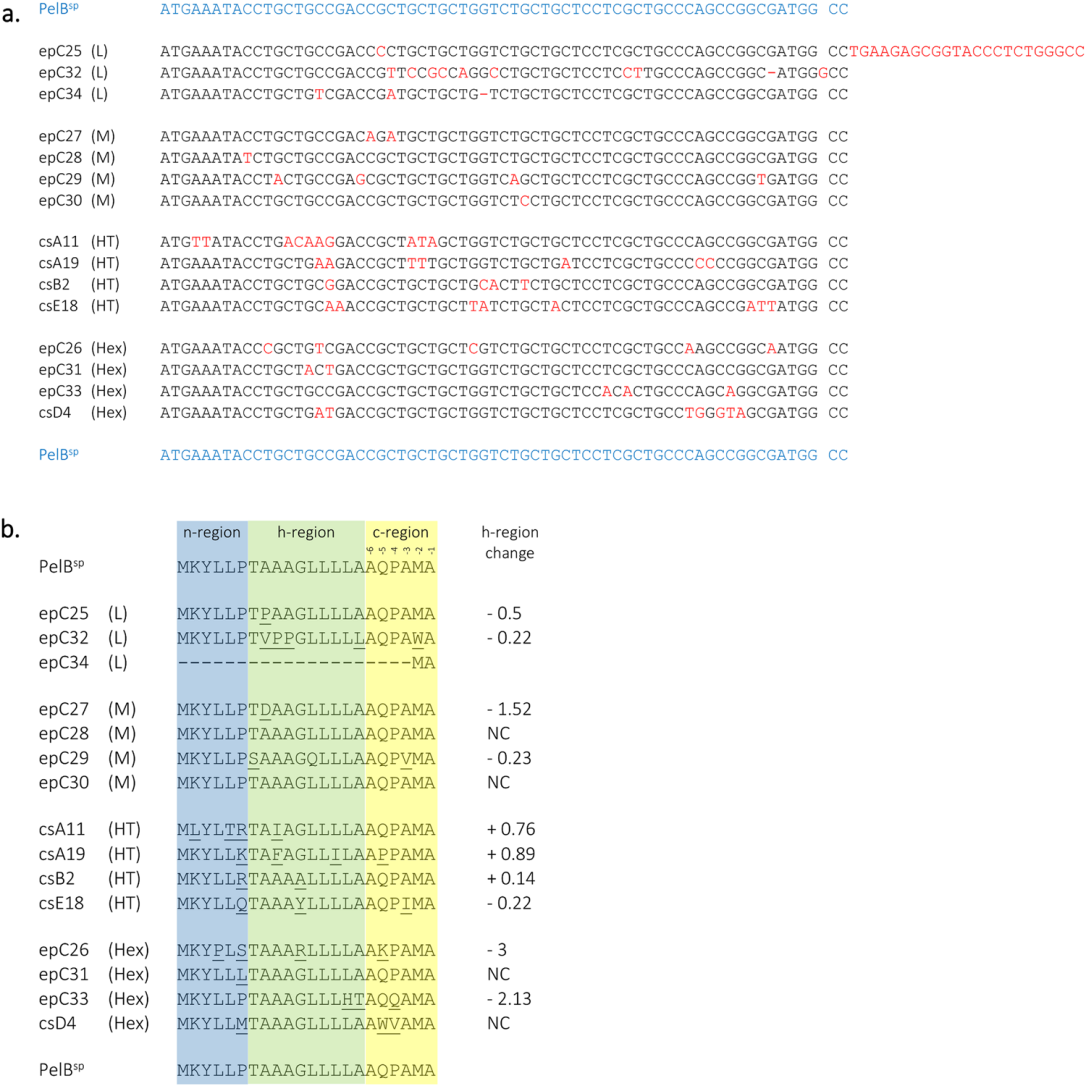
Sequence alignment of the signal peptides selected from epPCR and CS libraries. (**a**) Nucleotide sequence alignment; mutated bases are shown in red. L, M, HT and Hex refer to the β-lactamase activity conferred by the signal peptides: low, medium, high transporting and high expression respectively, see text for details. (**b**) Peptide sequence alignment. Signal peptides regions are highlighted. Underlined resides are those mutated. ‘h-region change’ states the change in hydrophobicity of the h-region, calculated using values from^34^. NC: no change.

To better understand the effect that the signal peptides evaluated above had on production and transport of scFv::Bla, they were sequenced (Fig. 6). Sequences are split into four categories: signal peptides giving rise to low (L) and medium (M) β-lactamase activity; those with high β-lactamase activity due to increased accumulation of scFv::Bla (high expression, Hex); and those with high β-lactamase activity due to enhanced partitioning of scFv::Bla to the periplasm (high transporting, HT).

Signal peptides targeting proteins to the SecB pathway comprise three regions: the N-terminal n-region, which has a positive charge thought to allow the signal peptide to associate with the negatively-charged inner membrane; a central h-region, which is hydrophobic and α helical in conformation and associates with SecA; and a C-terminal c-region, which has a β conformation^18,33^. The C-terminus of the c-region contains the highly conserved “AXA” motif (where X is any amino acid) that is recognised by signal peptidase I and is the site of signal peptide cleavage^33,34^.

Considering the low activity signal peptides, epC25 has an insertion and epC34 a deletion giving rise to a frameshift, resulting in deletion of the signal peptide. Signal peptide epC32 has a large number of nucleotide mutations giving rise to two additional proline residues (amino acids 9 and 10) in the h-region, likely to break the α-helical conformation of this region thereby inhibiting function. The four medium activity signal peptides analysed either have no amino acid changes in signal peptide sequence, or relatively small changes.

All four HT signal peptides have a mutation at amino acid 6 in the n-region, which is a proline in PelB^sp^. In signal peptides csA11, csA19 and csB2, this residue is mutated to a positively-charged amino acid; these three signal peptides gave rise to the highest partitioning of scFv::Bla into the periplasm (Fig. 5d). The mutations in csA19 and csB2 resulted in an increased overall positive charge of the n-region; this has been shown in previous studies to increase translocation^14^. The only sequenced signal peptides in this study with increased positive charge in the n-region are these two HT signal peptides. Signal peptide csA11 has an arginine residue at position 6, but position 2 is mutated from lysine to leucine, maintaining overall charge. Nonetheless, the position of charged residues as well as overall charge is known to be important in the n-region^14^. The fourth HT signal peptide (csE18) also has a mutation at proline 6, but to a glutamine. Three Hex signal peptides (epC26, epC31 and epD4) also have mutations at this position, although none to a charged amino acid. Taken together, this suggests that mutation of proline 6 significantly modulates signal peptide functionality. Future work will focus on the effect of specific mutations at this position in the signal peptide.

Within the h-region, three HT signal peptides (csA11, csA19 and csB2) have increased hydrophobicity when compared to PelB^sp^ (hydrophobicity values calculated according to^35^); the hydrophobicity of the h-region of these signal peptides correlates to the proportion of scFv::Bla in the periplasm (Fig. 5d). Numerous studies have found increased hydrophobicity in the h-region to increase protein translocation, although this is not always the case, and is dependent upon the protein being targeted to the periplasm and by the need to balance transport of recombinant proteins with native periplasmic proteins (reviewed by^36,37^). Again, none of the other sequenced signal peptides had increased hydrophobicity in the h-region, and two of the Hex signal peptides (epC26 and epC33) have dramatically lower hydrophobicity than PelB^sp^. Two HT signal peptides (csB2 and csE18) have mutations at glycine 11; glycine can act as a helix breaker, so these mutations could increase the helical nature of the h-region^36^. Again, one Hex signal peptide (epC26) also has a mutation at glycine 11, though combined with a dramatically decreased h-region hydrophobicity.

The relationship between the amino acid sequence of the c-region and function is not well understood, except for the consensus AXA motif which is recognised by signal peptidase I^34^. The HT signal peptides have few mutations in this region: csA19 has a proline in position −5, proline being a common amino acid in this region, thought to promote a β-turn^38^. Signal peptide csE18 has an isoleucine at position −3, breaking the AXA rule. Karamyshev *et al*.^38^ replaced alanine with leucine at position −3, resulting in a functional signal peptide, but did not attempt to insert isoleucine at this position; nonetheless, position −3 is less conserved than position −1^33,38^.

Three Hex signal peptides (epC26, epC33 and scD4) have mutations at positions −4 and −5 in relation to the peptidase cleavage site. It has been reported that medium-sized amino acids are preferred at positions −4 and −5^14,38^. The mutations at position −5 in epC26 and csD4 (lysine and tryptophan) are rare at this position in Gram negative bacterial signal peptides^38^. There is little information about the role of position −4, although the mutations in epC33 and epD4 at this position (glutamine and valine) are underrepresented in known signal peptides^38^.

In summary, signal peptide performance is thought to be determined by a combination of amino acid sequence attributes: the overall charge and position of charged residues in the n-region; the hydrophobicity and conformation of the h-region; and attributes of the c-region. These changes alter the way in which the signal peptide interacts with the membrane, Sec translocase and signal peptidase. The enhanced performance of the HT signal peptides csA11, csA19 and csB2 is likely due to a combination of increased and/or redistributed positive charge in the n-region and increased hydrophobicity of the h-region. However, the reasons for the enhanced performance of csE18 (although not as enhanced as csA11, csA19 and csB2) are unknown.

In addition to changes in amino acid sequence, codon usage can also affect signal peptide performance, changing translation rates and thereby folding and translocation^20,39^. Rare codons corresponding to <10% usage in *E*. *coli* B^40^ are present in three HT signal peptides: csA11 has arginine 6 encoded by AGG and isoleucine 9 by ATA; csA19 has proline 18 encoded by CCC; and csE18 has a silent mutation changing the codon encoding leucine 13 from CTG to CTA. However, some rare codons were also added to signal peptides in the low, medium and Hex groups, so codon bias is only one factor in determining signal peptide functionality.

The data presented here reflect the complexities of correlating amino acid sequence to signal peptide function, and confirm the need for screening to select improved signal peptides. The data also confirm that signal peptide amino acid sequence is not only important for interaction with the Sec apparatus and translocation, but also regulates translation of the recombinant protein.

### Performance of signal peptides in fed-batch fermentations for production of scFv::Bla

**Figure 7:**
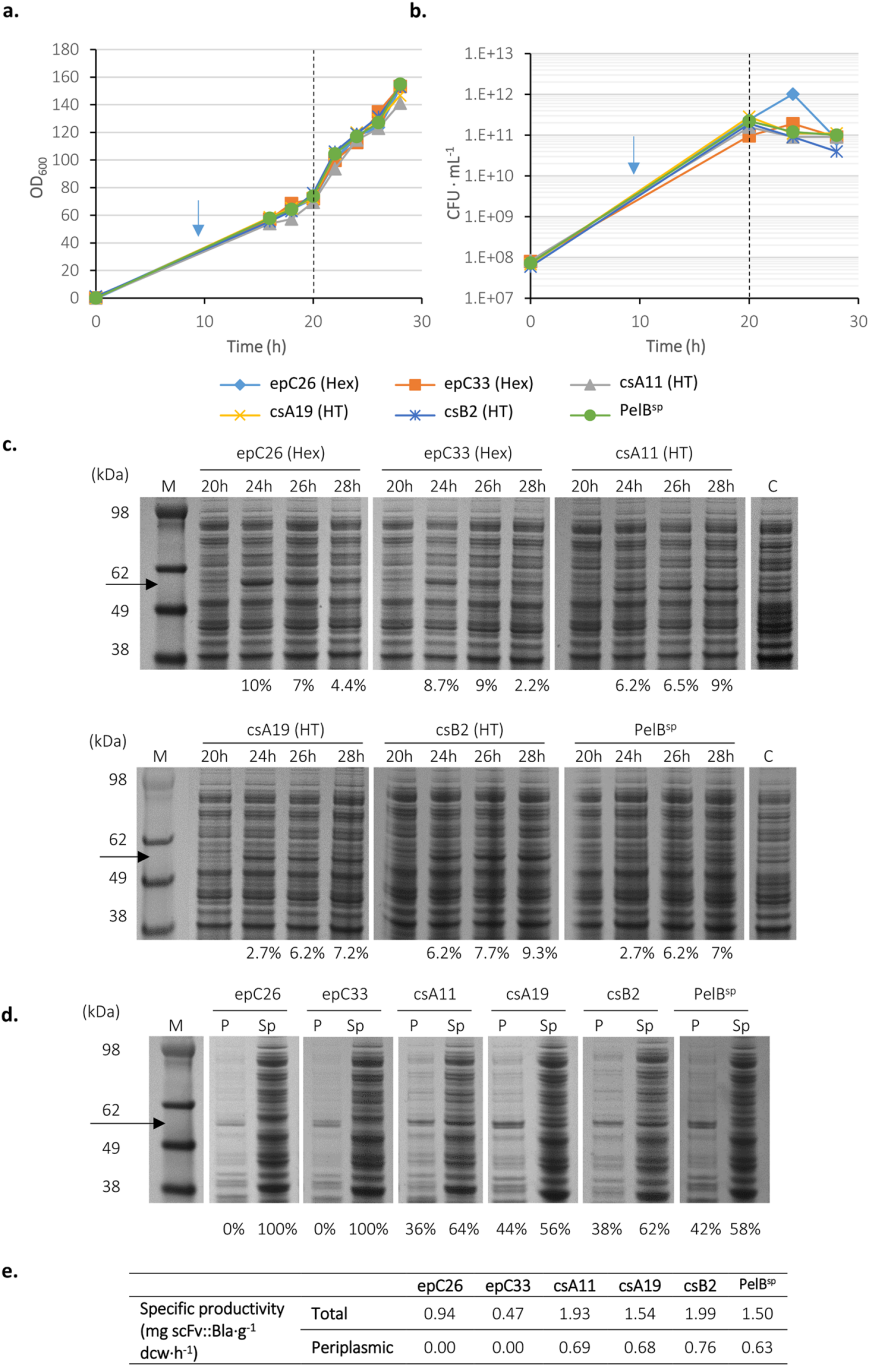
Fed-batch fermentation for the production of scFv::β-lactamase. *E*. *coli* BL21-A transformed with vectors encoding scFv::Bla with five high-activity signal peptides (Hex, high expression; HT, high transporting) from the epPCR and CS libraries or PelB^sp^ was grown in an Ambr^®^ 250 modular fermentation system at 30 °C. The feed was started after 10 hours (blue arrow) and cultures were induced with 0.02% arabinose at an OD_600_ ≈ 70–80 (20 h, dotted line) whereupon the temperature was changed to 25 °C. The OD_600_ **(a)** and CFU **(b)** were measured. **(c)** SDS-PAGE analysis of whole cell protein before (20 h) and after induction. Samples obtained from cultures carrying the scFv::Bla without the signal peptide (**c**) were used as a control. M is a molecular size marker. **(d)** Periplasm (P) and spheroplast (Sp) fractions from cultures after 28 hours of growth. The quantity of the scFv::Bla is expressed as percentage of whole cell protein (**c**) and the percentage of protein accumulated in each fraction (**d**). **(e)** Specific productivity of total scFv and periplasmic scFv at 28 hours growth. Values calculated from densitometry data from panels (**c**,**d**) as described in supplemental materials and methods. Uncropped gel images, and gel images for other time points, are available in Supplemental Fig. S16.

Five high activity signal peptides were evaluated in fed-batch high cell density fermentation cultures. Two high expression (epC26 and epC33) and three high transporting (csA11, csA19 and csB2) signal peptides were compared with PelB^sp^ for production and periplasmic transport of scFv::Bla using an Ambr^®^ 250 modular fermentation system. These five signal peptides were chosen on the basis of high β-lactamase activity, scFv::Bla accumulation and growth. A semi-defined medium and a glycerol-based feed using an exponential feeding profile to maintain µ = 0.1 were used^41^. Initial cultures were induced with arabinose at a low cell density (OD_600_ = 0.5); only epC26 and epC33, the two high expression clones, were able to achieve high cell density, the remaining cultures (including the PelB^sp^) displaying poor growth and culturability (Supplemental Fig. S8). Growth conditions were therefore optimised to permit growth of all cultures to high cell density; cultures were induced at a high cell density (OD_600_ between 70 and 80), with growth at 30 °C before induction and 25 °C afterwards. Growth and culturability were comparable for all cultures, with no growth defects observed following induction (Fig. 7a) although culturability decreased following induction, reflecting increased stress during RPP (Fig. 7b). Specific growth rates are compared in Table 2. SDS-PAGE analysis of whole cell lysates (Fig. 7c) showed that for high expression signal peptides epC26 and epC33, scFv::Bla accumulated at early stages of induction but by 28 hours (8 hours post-induction) had decreased, indicating loss of productivity, probably caused by plasmid loss or proteolysis. The accumulation of scFv::Bla (in terms of percentage of total cell protein) in these fed-batch cultures (maxima of 10% and 9% for epC26 and epC33) was far lower than in shake flasks (22% & 24% respectively; Fig. 4a). Fractionation (Fig. 7d) revealed that epC26 and epC33 did not direct scFv::Bla into the periplasm, corroborating shake flask experiments (Fig. 4b). Specific productivity data (Fig. 7e) reflect the above findings. Specific productivity was also lower for fed-batch fermenters than the corresponding shake flask experiments (Figs 4b and 5d).

**Table 2:** Specific growth rates for fed-batch fermentation cultures.

Time (h)	epC26	epC33	csA11	csA19	csB2	PelB^sp^
	scFv::Bla (Fig. 7)					
0						
16	0.391	0.403	0.393	0.430	0.239	0.420
18	0.061	0.092	0.029	0.054	0.064	0.053
20	0.055	0.024	0.096	0.053	0.092	0.070
22	0.158	0.164	0.150	0.182	0.167	0.173
24	0.087	0.060	0.100	0.069	0.058	0.057
26	0.022	0.090	0.036	0.036	0.050	0.042
28	0.107	0.064	0.069	0.065	0.075	0.099
**Time (h**)	**scFv** ** (Fig. ** **8** **)**					
0						
3	0.670	0.756	0.731	0.833	0.721	0.765
18	0.274	0.260	0.276	0.254	0.268	0.268
20	0.060	0.047	0.022	0.052	0.048	0.042
22	0.082	0.090	0.100	0.053	0.063	0.083
26	0.079	0.088	0.063	0.079	0.073	0.066
28	0.098	0.038	0.078	0.053	0.046	0.058
30	0.068	0.080	0.032	0.067	0.062	0.055

The high transporting signal peptides all showed equivalent or higher accumulation of scFv::Bla in whole cell lysates (between 7.2% and 9% of TCP) when compared to PelB^sp^ (7%; Fig. 7c), although (as with the high expression signal peptides) lower accumulation than in shake flask cultures (11–19%; Fig. 5c). Taken together with the data from the high expression clones, this suggests a general decrease in RP expression levels in the fed-batch fermentation system, probably due to increased metabolic requirements of high cell density culture. Given that fed-batch cultures induced with arabinose at an OD_600_ of 0.5 displayed growth defects (Supplemental Fig. S8), this also suggests that fed-batch growth needs to be carefully optimised in terms of titration of inducer concentration and induction point to balance not only metabolic requirements of growth and RPP but also potentially balance throughput of RP and native proteins through the Sec translocase. Fractionation revealed that a comparable proportion of scFv::Bla was present in the periplasm for the high transporting clones (36–44%) and PelB^sp^ (42%; Fig. 7d). However, comparison of shake flask fractionation data (Fig. 5d) with fed-batch data (Fig. 7d) reveals that PelB^sp^ was able to direct far more scFv::Bla into the periplasm at high compared to low cell density (42% versus 10%). Again, this probably reflects the balance between pBAD promoter strength and RP versus native protein transport through Sec.

### Fed-batch production of scFv without a β-lactamase fusion

The *bla* gene was removed from each plasmid construct assessed in Fig. 7 and fed-batch fermentation was repeated. Although growth and culturability were good (Supplemental Fig. S9a,b), scFv did not accumulate to high levels; Western blotting was needed to detect scFv on SDS-PAGE gels (Supplemental Fig. S9c,d). High expression signal peptide epC26 accumulated scFv mainly in a precursor form in the spheroplast fraction; epC33 permitted slightly better transport to the periplasm and signal peptide cleavage. All CS library signal peptides allowed good partitioning to the periplasm and signal peptide cleavage. The scFv was also detected in samples obtained from the culture medium.

**Figure 8:**
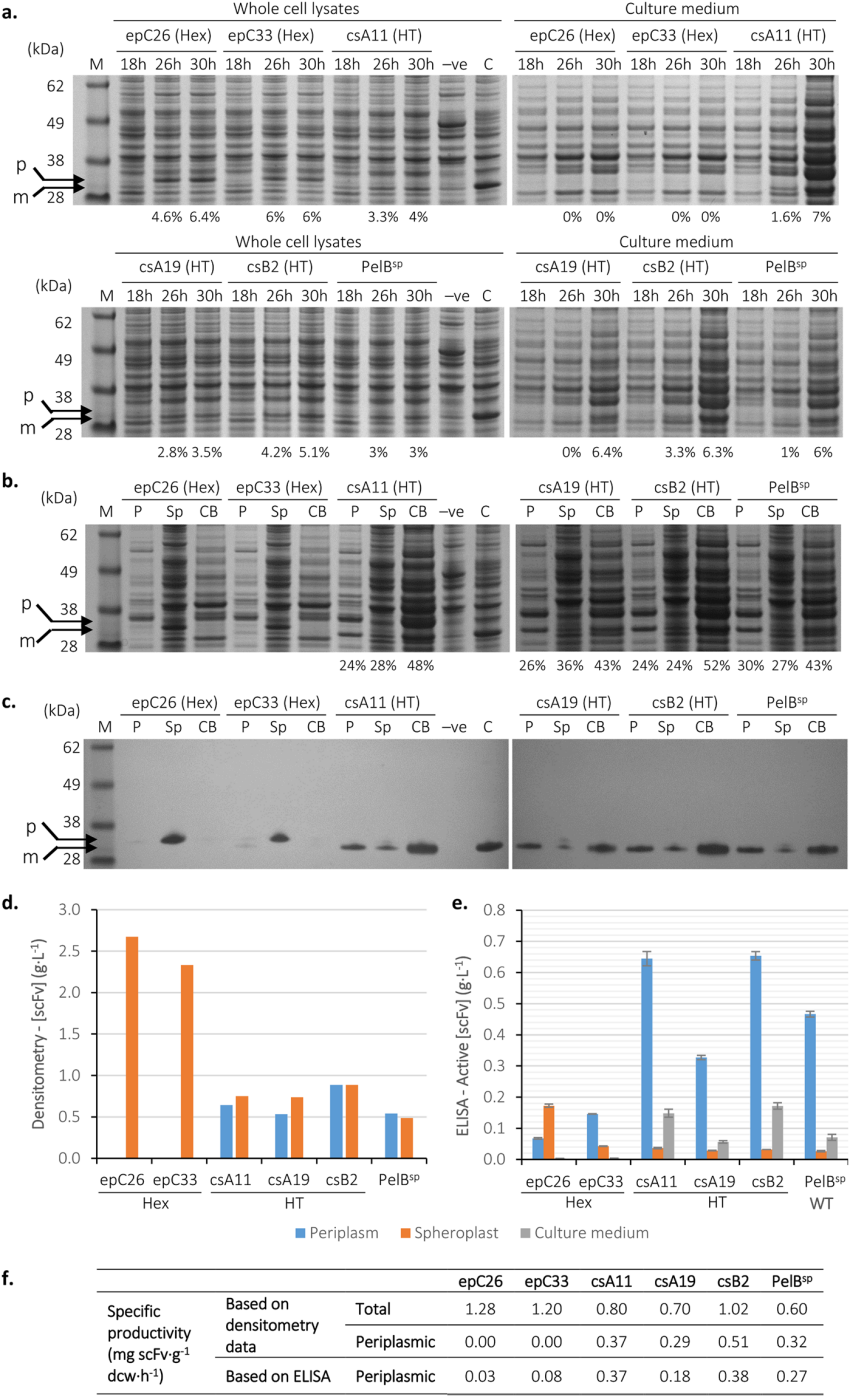
Production of scFv by fed-batch fermentation induced with 0.2% arabinose. *E*. *coli* BL21-A transformed with vectors encoding scFv with five high-activity signal peptides or PelB^sp^ was grown in an Ambr®250 modular fermentation system at 30 °C. Cultures were induced with 0.2% arabinose at an OD_600_ ≈ 70–80 whereupon the temperature was changed to 25 °C. (**a**) Whole cell lysates and culture broth were analysed by SDS-PAGE before (18 h) and after induction (26 h and 30 h). Controls are 24 hour cultures of empty vector (−ve) and cytoplasmic scFv (C). M is a molecular size marker. Periplasmic, spheroplast and culture broth fractions from 30 hours growth were analysed by SDS-PAGE (**b**) and Western blotting using anti-myc (**c**). Arrows show precursor (p) and mature (m) scFv, as determined by comparison of molecular weight. The quantity of scFv is expressed as percentage of whole cell or culture medium protein (**a**) or percentage in each fraction (**b**) at the bottom of the gel. The amount of scFv was determined using densitometry analysis of the SDS-PAGE gels (**d**) and active scFv was determined using ELISA (**e**). Uncropped gel images are available in Supplemental Fig. S17. (**f**) Specific productivity data based on (d) and (e).

As high levels of scFv did not accumulate under these growth conditions, the fermentation was repeated with induction using 0.2% arabinose. In comparison to the fed-batch experiment with induction using the lower arabinose concentration, growth was similar (growth curves and CFU data in Supplemental Fig. S10; specific growth rates in Table 2) but accumulation of scFv was far higher (Fig. 8a), visualisation of scFv being possible by SDS-PAGE. As with the scFv::Bla fusion, the Hex signal peptides accumulated more scFv than the HT signal peptides (6–6.4% of TCP at 30 h versus 3.5–5.1%), but very little was transported to the periplasm (Fig. 8c). However, scFv accumulation under these conditions was still lower than accumulation of scFv::Bla during fed-batch growth and 0.02% arabinose induction (up to 10%; Fig. 7c). This decrease in scFv accumulation when Bla is not present could indicate that Bla acts to increase the efficiency of translation or transport, or enhance the stability of scFv during synthesis and transport to the periplasm. This is a similar principle to the use of periplasmic fusion tags such as maltose binding protein (MBP) or DsbA, although these fusions tend to be at the N-terminal of the recombinant protein of interest^42,43^.

Culture medium samples revealed that scFv leaked from cells with the HT signal peptides and PelB^sp^. Analysis of fractions confirmed transport of scFv to the periplasm by these signal peptides (Fig. 8b,c). As Hex signal peptides epC26 and epC33 poorly translocated scFv to the periplasm, there was no leakage.

Finally, scFv activity was assayed using ELISA (Fig. 8e) and compared with densitometry analysis of SDS-PAGE gels (Fig. 8d). As expected, epC26 and epC33 samples had relatively low scFv activity as determined by ELISA; epC33, having better translocation of scFv than epC26, had a higher periplasmic activity. The relatively high activity of the spheroplast fraction in epC26 probably reflects scFv activity in the absence of disulphide bonding. Although a disulphide bond is required for correct folding of Fv domains^4^, scFv 13R4 is an engineered scFv selected for enhanced folding in the cytoplasm of *E*. *coli* where disulphide bonding is not achieved^24^. Both high expression signal peptides (epC26 and epC33) gave rise to precursor scFv in the spheroplast fraction, (Fig. 8c,d) indicative of cytoplasmic accumulation.

Comparing the HT signal peptides with PelB^sp^, csA11 and csB2 both had higher periplasmic and culture medium scFv activity and higher [scFv] as determined by densitometry than the wild type PelB^sp^; periplasmic scFv activities from csA11 and csB2 cultures were around 40% higher than PelB^sp^. HT signal peptide csA19 had lower performance than PelB^sp^ in terms of active scFv as determined by ELISA, and comparable [scFv] as determined by SDS-PAGE. Specific productivity data, based both on the scFv concentration measured using SDS-PAGE and ELISA, (Fig. 8f) confirm these observations, with csA11 and csB2 having higher specific productivity than PelB^sp^. The decrease in specific productivity between scFv::Bla (Fig. 7e) and scFv (Fig. 8f) can be explained in part by the smaller molecular mass of scFv compared to the scFv::Bla fusion. Growth of cultures containing csA11 and csB2 was comparable to PelB^sp^ (Supplemental Fig. S10), indicating suitability for scale-up. We can therefore confirm that the screening system has successfully generated signal peptides with better performance than PelB^sp^ for the periplasmic production of this scFv. Peak productivity was determined as 0.65 g scFv·L^−1^ in the periplasmic fraction. Despite the drop in scFv accumulation when compared to scFv::Bla, the screening system nonetheless generated signal peptides capable of better production of scFv 13R4 than PelB^sp^. Future work should focus on further development of the system, including fermentation development to optimise process conditions and minimise the quantity of scFv in the culture medium for selected signal peptides^17^.

## Conclusions

Selection of signal peptides for translocation of recombinant proteins to the *E*. *coli* periplasm remains an important tool for bioprocessing, especially with the development of novel antibody fragment therapeutics. If *E*. *coli* is going to compete with CHO cells as an expression system for antibody fragments, platform technology (similar to the CHO platforms for monoclonal antibody production^44^) needs to be developed. Selection of signal peptides is an important part of this platform development: we have shown that signal peptide sequence not only affects translocation but also production of scFv 13R4. This study has demonstrated for the first time the utility of C-terminal β-lactamase fusions for screening signal peptides for the translocation of recombinant proteins to the periplasm. Screening remains an essential step in signal peptide development due to the non-generic nature of signal peptides^14^; the methods developed here will assist in this process. We have also demonstrated that, although some signal peptides increased translation but not translocation of scFv::Bla, and overall expression levels of scFv 13R4 were lower when the β-lactamase fusion was removed, two signal peptides that generated elevated β-lactamase activities in the scFv::Bla screen (csA11 and csB2) were able to improve production of periplasmic scFv 13R4 by ~40% (comparing active scFv as determined by ELISA) compared to PelB^sp^. Specific productivities of csA11 and csB2 were also greater than that of PelB^sp^. We propose that a workflow comprising a β-lactamase activity screen followed by assessment of high activity clones using growth and analysis of periplasmic protein content is a good approach to develop improved production strains for antibody fragments or other periplasmically-targeted recombinant proteins.

## Materials and Methods

### Strains and plasmids

*E*. *coli* strains used were the expression strain BL21-A, derived from *E*. *coli* B (*F*^*−*^ *ompT gal dcm lon hsdSB (rB*^*−*^*mB*^*−*^*) [malB*^+^*]K-12(λS) Δ(ara)*) sourced from Cobra Biologics, and the electrocompetent ElectroSHOX sourced from Bioline, UK (F^-^ *mcr*A Δ(*mrr*-*hsd*RMS-*mcr*BC) Φ80*lac*Z ΔM15 Δ*lac*X74 *rec*A1 *end*A1 *ara* Δ139 Δ(*ara*, *leu*)7697 *gal*U *gal*K λ^−^ *rps*L (Str^R^) *nup*G), which was used for cloning. Plasmids are detailed in Supplemental Table S1 and PCR and other oligonucleotide primers in Supplemental Table S2.

### Growth media, antibiotics and standard solutions

Lennox broth (LB) contained 10 g·L^−1^ BBL^TM^ phytone peptone (BD), 5 g·L^−1^ Bacto^TM^ yeast extract (BD) and 5 g·L^−1^ NaCl in deionised water. Terrific broth (TB) was prepared by dissolving 47 g·L^−1^ of terrific broth powder (Thermo Fisher Scientific) and 4 mL·L^−1^ of glycerol in deionised water. Lennox agar contained 10 g·L^−1^ BBL^TM^ phytone peptone, 5 g·L^−1^ Bacto^TM^ yeast extract, 5 g·L^−1^ NaCl and 15 g·L^−1^ extra pure agar. Mueller-Hinton (M-H) agar (Sigma-Aldrich) contained 38 g·L^−1^ of premade Mueller-Hinton agar powder in deionised water. Kanamycin (Sigma-Aldrich) was routinely used at a concentration of 50 µg·mL^−1^ and ampicillin (Sigma-Aldrich) at 100 µg·mL^−1^. Phosphate Buffered Saline (PBS) contained 8 g·L^−1^ NaCl, 0.2 g·L^−1^ KCl, 2.16 Na_2_HPO_4_·7H_2_O and 0.2 g·L^−1^ KH_2_PO_4_ in deionised water, adjusted to pH 7.2–7.4.

### SDS-PAGE, Western blotting and ELISA

Details can be found in the Supplementary methods section.

### Purification of scFv 13R4 for ELISA standards

Reference scFv 13R4 was generated using a fed-batch fermentation of *E*. *coli* BL21-A carrying pLBAD2-PelB-scFv13R4 using growth conditions as described in Supplemental Fig. S10. Culture medium samples were taken (this fraction only contained mature scFv 13R4), filtered through a 0.2 µm filter and pretreated with 40 mM imidazole and 500 mM NaCl. A 1 mL nickel Sepharose HisTrap FF column (GE Healthcare) was washed with 5 column volumes of deionised water then 5 column volumes of binding buffer (PBS pH 7.2, 40 mM imidazole, 500 mM NaCl). The column was loaded with 10 mL of culture sample, washed with 15 column volumes of binding buffer, and eluted with 5 column volumes of elution buffer A (PBS pH 7.2, 100 mM imidazole, 500 mM NaCl) then 5 column volumes of elution buffer B (PBS pH 7.2, 500 mM imidazole, 500 mM NaCl). Eluted fractions were analysed by SDS-PAGE (Supplemental Fig. S11).

### Subcellular fractionation

A modified cold osmotic shock fractionation procedure was used^9^ except for data shown in Fig. 1. Cell pellets (equivalent to 1 OD_600_·mL) were suspended in 50 µL of ice-cold spheroplast buffer (100 mM Tris-HCl pH 8.2, 500 mM sucrose, 5 mM EDTA) then lysozyme was added to 0.8 mg·mL^−1^ followed by 50 µL of ice-cold deionised water, and incubated on ice for 5 min. MgSO_4_ was added to a concentration of 20 mM then the samples were centrifuged (12 000 *g*, 2 min). The supernatant was the periplasmic fraction, the pellet (resuspended in 100 µL of 100 mM Tris-HCl pH 8) was the spheroplast fraction.

For the data in Fig. 1, a cold osmotic shock fractionation procedure was used. Cell pellets (equivalent to 1 OD_600_·mL) were resupended in 150 μL of ice-cold spheroplast buffer (100 mM Tris-HCl pH 8, 500 mM sucrose, 0.5 mM EDTA) and stored on ice for 5 min. Fifty microliters were transferred to a new centrifuge tube, constituting the whole lysate protein sample. The cell suspension was centrifuged at 12 000 *g* for 1 minute. The supernatant was discarded and the pellet was resuspended in 100 μL of ice-cold deionised water. Ice-cold MgCl_2_ was added to a final concentration of 20 mM, the cell suspension was centrifuged at 12 000 *g* for 2 minutes. The supernatant was removed, comprising the periplasmic protein fraction, and the pellet was resuspended in 100 μL of 10 mM Tris-HCl pH 8, comprising the spheroplast protein fraction.

### Cloning & screening

Details of cloning and methods can be found in the Supplementary methods section.

### MIC determination

A single colony was used to inoculate 10 mL of LB plus 50 µg·mL^−1^ kanamycin, which was grown at 37 °C to an OD_600_ of 1–2, then serially diluted in sterile PBS to a concentration of 10^5^–10^8^ CFU·mL^−1^. Dilutions were plated onto Mueller-Hinton agar supplemented with 50 µg·mL^−1^ kanamycin, 0.2% or 0.02% arabinose and 3–1600 µg·mL^−1^ ampicillin. Plates were incubated at 37 °C or 25 °C.

### Beta lactamase assay

β-lactamase activities of whole cells were measured according to Angus *et al*.^23^ and O’Callaghan^30^. Cells were grown in 96-well plates: cells from a colony were added to 200 µL of TB supplemented with 50 µg·mL^−1^ kanamycin in each well and grown at 25 °C overnight. Ten µL of each culture was added to each well of a fresh 96-well plate containing 200 µL TB supplemented with 50 μg · mL^−1^ of kanamycin, which was grown at 25 °C for 2 h whereupon arabinose was added to 0.02% to induce RPP. At time intervals, 96-well plates were centrifuged (1 218 *g*, 15 min) and pellets resuspended in 10 mM Na-HEPES pH 7.0 and 100 µL of nitrocefin working solution (1 mg·mL^−1^ nitrocefin (Carbosynth, Reading, UK) in 200 µL DMSO and 9.8 mL Na-HEPES pH 7.0) was added to each well. The OD_495_ was measured continuously for 30 minutes; reaction rates were normalised by division by OD_600_ (as a measure of biomass concentration) and are in units of change in OD_495_ per minute per OD_600_. The initial reaction velocity was calculated by linear regression and adapted to obtain an R ≥ 0.9 (where possible) using GraphPad Prism 7 software. The 5% uncertainty of the prediction of the slopes was also calculated and plotted as error bars.

### DNA sequencing and protein sequencing

Vector modifications were confirmed by sequencing. DNA sequencing was carried out by Genewiz, formerly Beckman Coulter Genomics, with the exception of the sequencing of signal peptide libraries, which was carried out by Eurofins Genomics.

### Fermentations using Ambr^®^ 250 modular fermentation system

Starter cultures were grown in 10 mL of LB with 50 µg·mL^−1^ kanamycin at 25 °C to an OD_600_ of 2, where they were added to 50 mL of LB with 50 µg·mL^−1^ kanamycin in a 250 mL baffled shake flask which was incubated at 25 °C at 200 rpm until an OD_600_ of between 4 and 6.

Fed-batch fermentations used the Ambr^®^ 250 modular fermentation system (TAP Biosystems, Sartorius Stedim) which comprises 250 mL single-use bioreactors. Fermentations started with 150 mL of batch medium and 100 mL of feed. The batch medium was from Want *et al*.^41^ and comprised batch salts (14 g·L^−1^ (NH_4_)_2_SO_4_, 35 g·L^−1^ glycerol, 20 g·L^−1^ Bacto^TM^ yeast extract, 2 g·L^−1^ KH_2_PO_4_, 16.5 g·L^−1^ K_2_HPO_4_, 7.5 g·L^−1^ citric acid, 1.5 mL · L−^1^ concentrated H_3_PO_4_ and 0.66 mL·L^−1^ PPG 2 000) and additions (34 mL·L^−1^ trace elements solution (comprising 3.36 g·L^−1^ FeSO_4_·7H_2_O, 0.84 g·L^−1^ ZnSO_4_·7H_2_O, 0.15 g·L^−1^ MnSO_4_·H_2_O, 0.25 g·L^−1^ Na_2_MoO_4_·2H_2_O, 0.12 g·L^−1^ CuSO_4_·5H_2_O, 0.36 g·L^−1^ H_3_BO_3_ and 48 mL·L^−1^ concentrated H_3_PO_4_), 10 mL·L^−1^ 1 M MgSO_4_·7H_2_O, 2 mL·L^−1^ 1 M CaCl_2_·2H_2_O and 1 mL·L^−1^ 50 mg·mL^−1^ kanamycin stock). The feed solution comprised 714 g·L^−1^ glycerol, 30 mL·L^−1^ 1 M MgSO_4_·7H_2_O, and 1 mL·L^−1^ 50 mg·mL^−1^ kanamycin. All culture medium components were filter sterilised and added to the fermentation vessels before use.

The pH was maintained at 7.0 using 10% NH_4_OH and 1 M HCl. Polypropylene glycol (PPG 2 000) was used as antifoam. The dissolved oxygen was maintained at above 20% using cascade control (increasing the stirrer speed followed by addition of O_2_). Bioreactors were inoculated to an OD_600_ of 0.1. Exponential feeding was used according to equation 1.

1 $$F=(\frac{1}{S})\times (\frac{\mu }{{Y}_{XS}}+m)\times {X}_{0}\times {e}^{\mu t}$$

where *F* is the feed rate in L·h^−1^, *S* is the substrate concentration in the feed (714 g·L^−1^), *μ* is the required specific growth rate (0.1 h^−1^), *Y*_*XS*_ is the yield coefficient (0.4 g biomass per g glycerol), *m* is the maintenance coefficient (0.04), *X*_0_ is the biomass in g at the start of the feed and *t* is time. The feed was started when the DO increased, indicating nutrient limitation.

### Data availability

All data generated or analysed during this study are included in this published article and its supplementary information files.

## Electronic supplementary material

Supplementary information
