# Supplementary material for: Development of a generic β-lactamase screening system for improved signal peptides for periplasmic targeting of recombinant proteins in Escherichia coli
Source: Sci Rep. 2018 May 3;8:6986. doi: 10.1038/s41598-018-25192-3 (PMC5934370; doi:10.1038/s41598-018-25192-3)
Supplement: Supplementary file 1 — Supplementary information [file 41598_2018_25192_MOESM1_ESM.pdf]

Supplemental information

Development of a generic β-lactamase screening system for improved signal peptides for periplasmic targeting of recombinant proteins in *Escherichia coli*

T Selas Castiñeiras[1,2,3], SG Williams[1], A Hitchcock[1], JA Cole[3,4], DC Smith[1], TW Overton[2,3*].

[1]Cobra Biologics, Stephenson Building, The Science Park, Keele ST5 5SP, UK.

[2]School of Chemical Engineering, [3]Institute of Microbiology & Infection, and [4]School of Biosciences, The University of Birmingham, Edgbaston, Birmingham B15 2TT, UK

Supplemental figures S1-S11 P2-16

Supplemental tables S1-S2 P17-18

Supplemental materials and methods P19-24

Supplemental figures S12-S17 P27-35

(uncropped images of SDS-PAGE gels and Western blots)

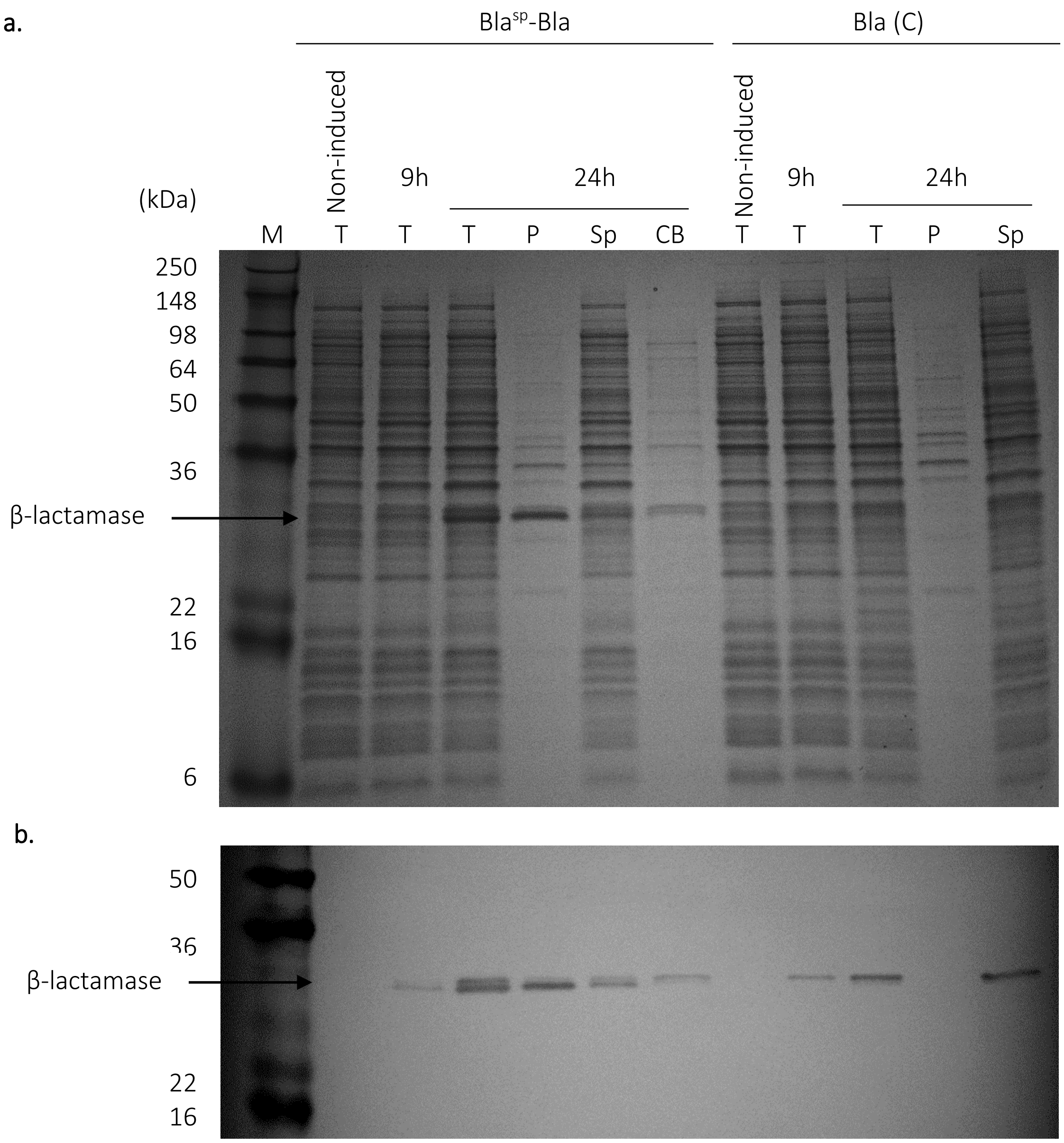

Supplemental Figure S1. Evaluation of the production of β-lactamase. Cultures containing plasmids expressing β-lactamase with or without its own signal peptide were grown at 25 °C and induced with 0.02% of arabinose at an OD_{600} ≈ 0.5; growth and CFU data are shown in Fig. 2b&c. Samples were taken after 9 and 24 hours growth, periplasmic (P) and spheroplast (Sp) fractions were separated, and proteins were analysed by SDS-PAGE **(a)** and Western blotting using anti-Bla **(b)**.T = total cell protein, CB = culture broth, M = molecular size marker. Only the mature Bla, in reduced and oxidised forms, can be observed in the samples.

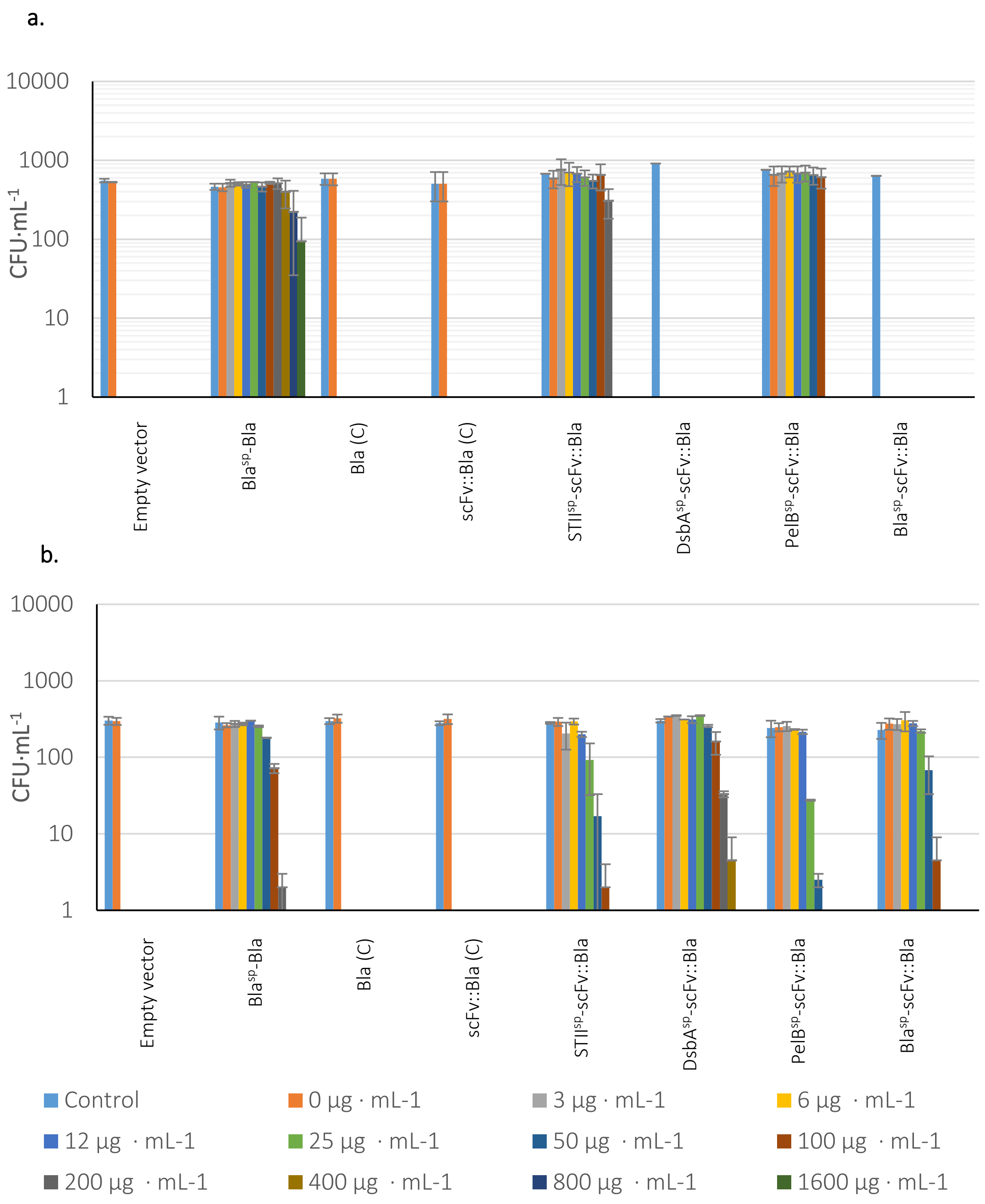

Supplemental Figure S2. Minimum inhibitory concentration (MIC) evaluation of the production of β-lactamase and scFv::β-lactamase at **(a)** 37 °C or **(b)** 25 °C. *E. coli* BL21-A carrying plasmids as detailed in Fig. 2a were grown in TB at 37 °C (a) or 25 °C (b). Samples were serially diluted and plated onto Mueller-Hinton (M-H) agar containing 50 μg·mL^{-1} kanamycin, 0.2% arabinose (a) or 0.02% arabinose (b) and concentrations of ampicillin from 3 to 1600 μg·mL^{-1}. An M-H agar plate supplemented only 50 μg·mL^{-1} kanamycin was used as control. M-H agar plates were incubated at 37 °C for 12 - 18 hours (a) or 25 °C for 48 hours (b). Data are shown as mean values from two independent experiments, error bars are ±1 standard deviation.

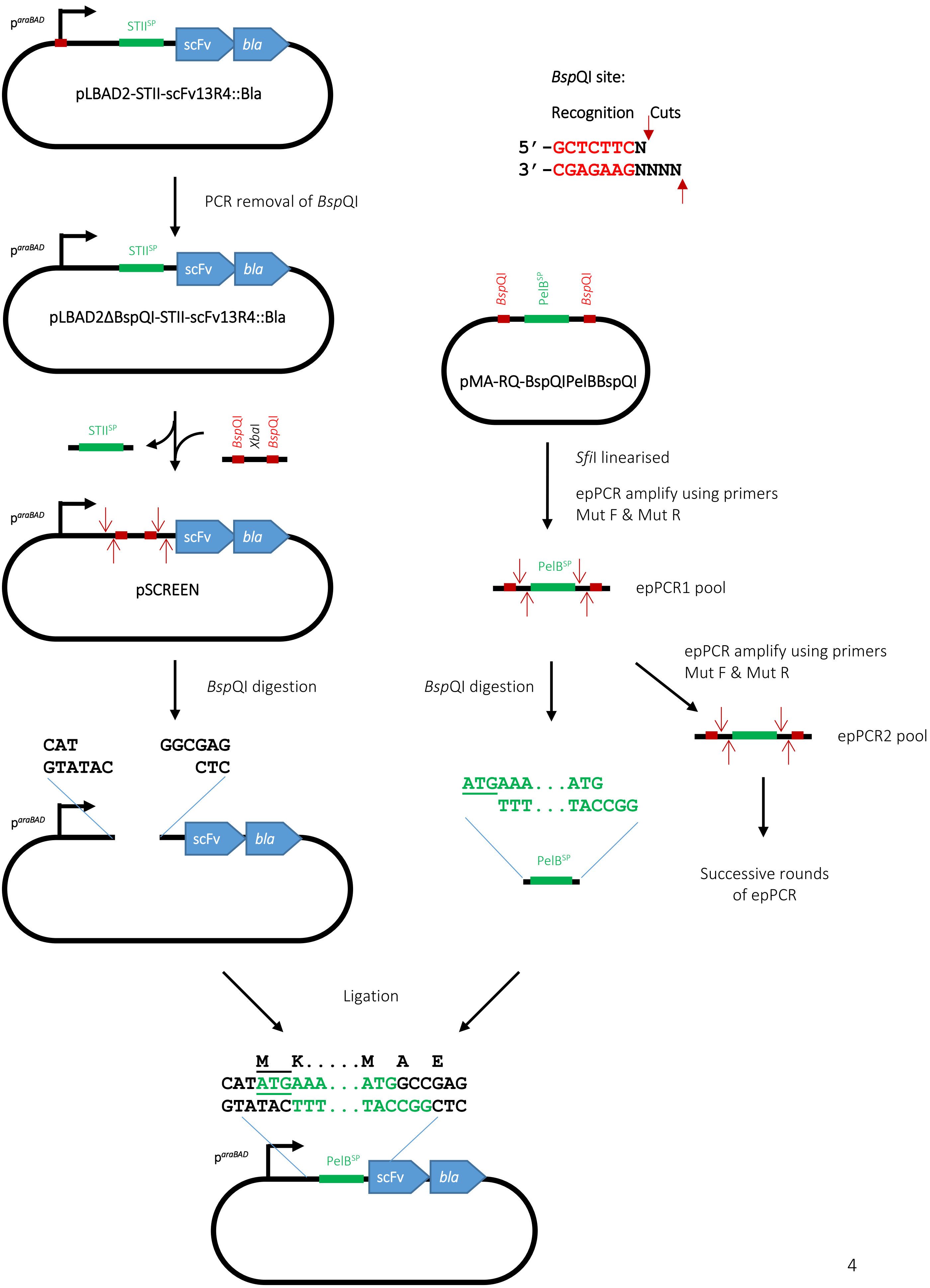

p araBAD
STII SP
scFv
bla
pLBAD2-STII-scFv13R4::Bla
PCR removal of BspQI
pLBAD2ΔBspQI-STII-scFv13R4::Bla
BspQI
XbaI
BspQI
pSCREEN
BspQI digestion
CAT
GTATAC
GGCGAG
CTC
BspQI site:
Recognition
Cuts
5′ -GCTCTTCN
3′ -CGAGAAGNNNN
PelB SP
pMA-RQ-BspQIPelBBspQI
SfiI linearised
epPCR amplify using primers
Mut F & Mut R
epPCR1 pool
epPCR2 pool
Successive rounds
of epPCR
ATGAAA...ATG
TTT...TACCGG
Ligation
M K.....M A E
CATATGAAA...ATGGCCGAG
GTATACTTT...TACCGGCTC

Supplemental Figure S3. Cloning strategy for the signal peptide screen.

The screening plasmid pSCREEN was constructed in two steps from pLBAD2-STII-scFv13R4::Bla: PCR removal of a *Bsp*QI site; and replacement of the STII^{sp} with a *Bsp*QI-*Xba*I-*Bsp*QI linker. The error-prone PCR library was generated by amplifying PelB^{sp} from the pMA-RQ-BspQIPelBBspQI vector by epPCR using the Mut F and Mut R primers (top right). The epPCR pools 1-4 refer to 1-4 successive rounds of epPCR to increase the number of mutations per fragment. *Bsp*QI digestion of pSCREEN (recognition sites are shown as red boxes, cut sites as red arrows) and the epPCR PelB^{sp} fragment resulted in complementary overhangs, which were ligated.

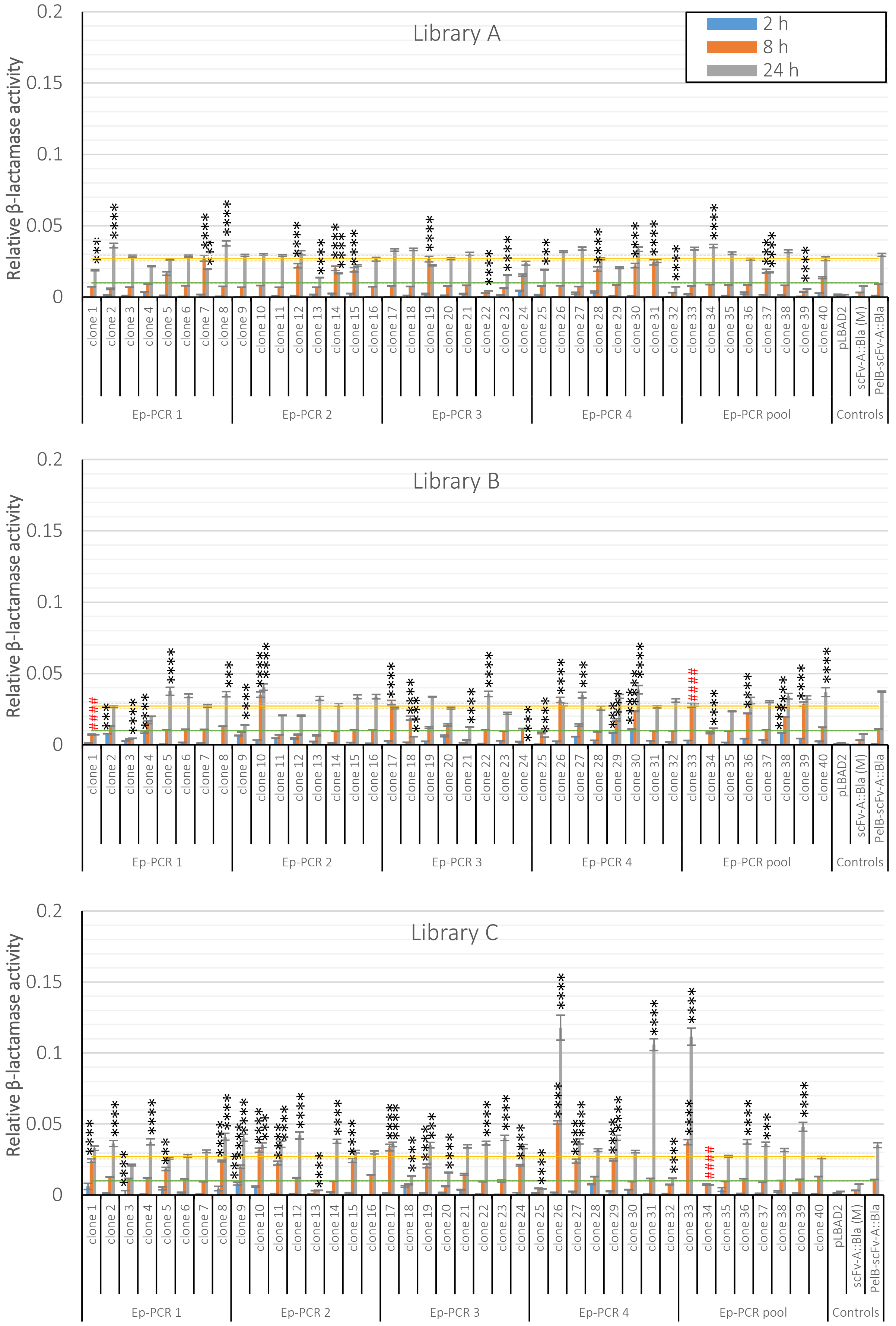

Library A
Library B
Library C
Relative β-lactamase activity
0.2
0.15
0.1
0.05
0
2 h
8 h
24 h
Ep-PCR 1
Ep-PCR 2
Ep-PCR 3
Ep-PCR 4
Ep-PCR pool
Controls
pLBAD2
scFv-A::Bla (M)
PelB-scFv-A::Bla

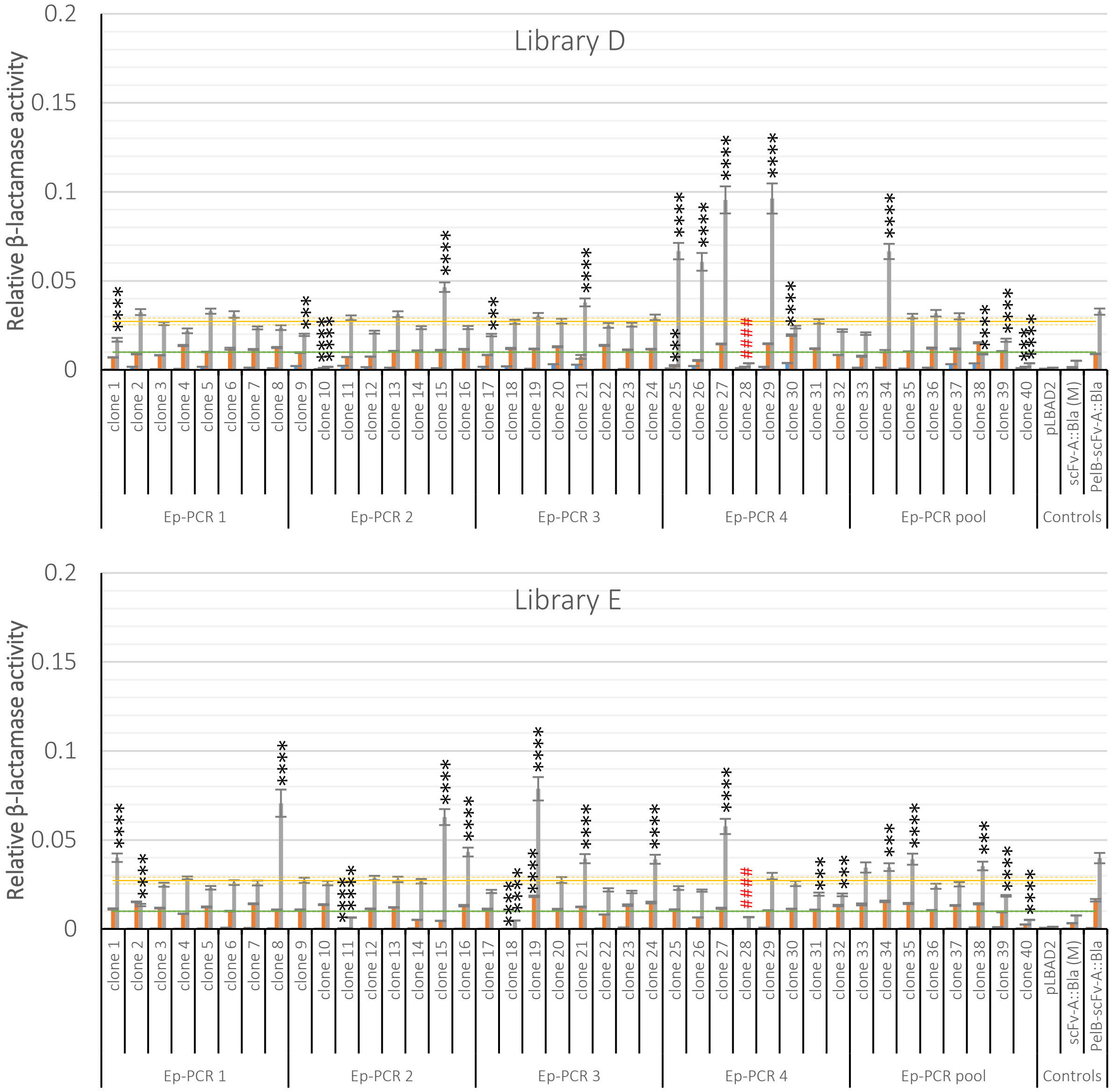

Supplemental Figure S4. β-lactamase activity screen of epPCR signal peptide library. *E. coli* BL21-A transformed with pSCREEN clones containing the error-prone PCR library signal peptides fused to scFv::*bla* were grown at 25 °C and induced with 0.02% arabinose after 2 hours growth. β-lactamase activity of cultures was measured by hydrolysis of nitrocefin before (2 h) and after induction (8 h and 24 h). The average β-lactamase activity of PelB^{sp} at 8 and 24 hours is shown by the green and yellow horizontal lines respectively. Error bars represent the 5% uncertainty in the calculation of the slopes corresponding to β-lactamase activity (n=2), which is expressed in terms of change in OD_{495} per minute per OD_{600}. Asterices indicate values statistically significant compared to the control (PelB^{sp}-scFv::Bla) after selecting adjusted $p < 0.001$ as the level of significance during statistical analysis of data. **** $p < 0.0001$; *** $0.001 > p > 0.0001$. #Indicates data excluded from the statistical analysis as failed the normality test after selecting $p<0.05$ as the level of significance (n=200).

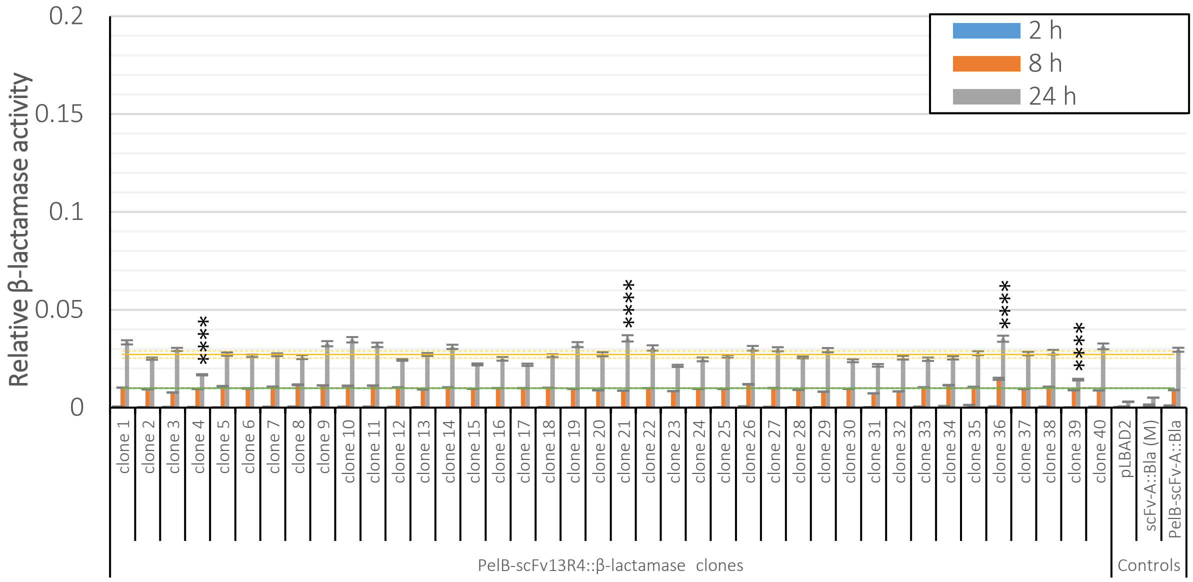

Supplemental Figure S5. Evaluation of colony to colony β-lactamase activity variability using the PelB signal peptide. *E. coli* BL21-A transformed with pLBAD2-PelB-scFv13R4::Bla were grown at 25 °C and induced with 0.02% arabinose after 2 hours growth. β-lactamase activity of cultures was measured by hydrolysis of nitrocefin before (2 h) and after induction (8 h and 24 h). The average β-lactamase activity of PelB^{sp} at 8 and 24 hours is shown by the green and yellow horizontal lines respectively. Error bars represent the 5% uncertainty in the calculation of the slopes corresponding to β-lactamase activity (n=2), which is expressed in terms of change in OD_{495} per minute per OD_{600}. **** Indicates values statistically significant ($p < 0.0001$) compared to the control (PelB^{sp}-scFv::Bla) after selecting adjusted $p < 0.001$ as the level of significance during statistical analysis of data (n=40).

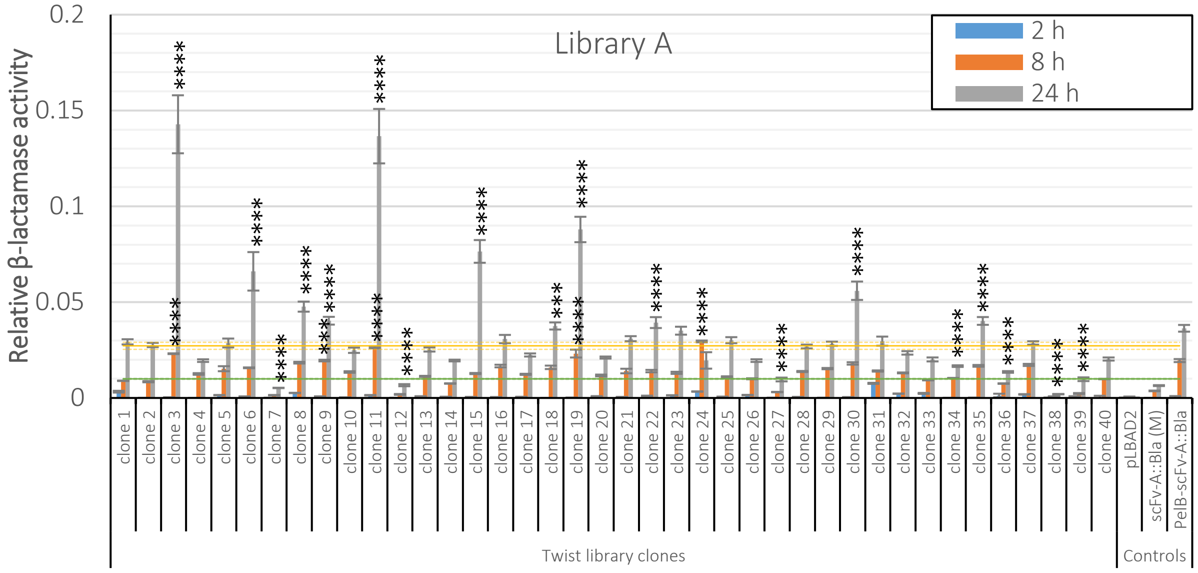
Library A
2 h
8 h
24 h
Relative β-lactamase activity
Twist library clones
Controls

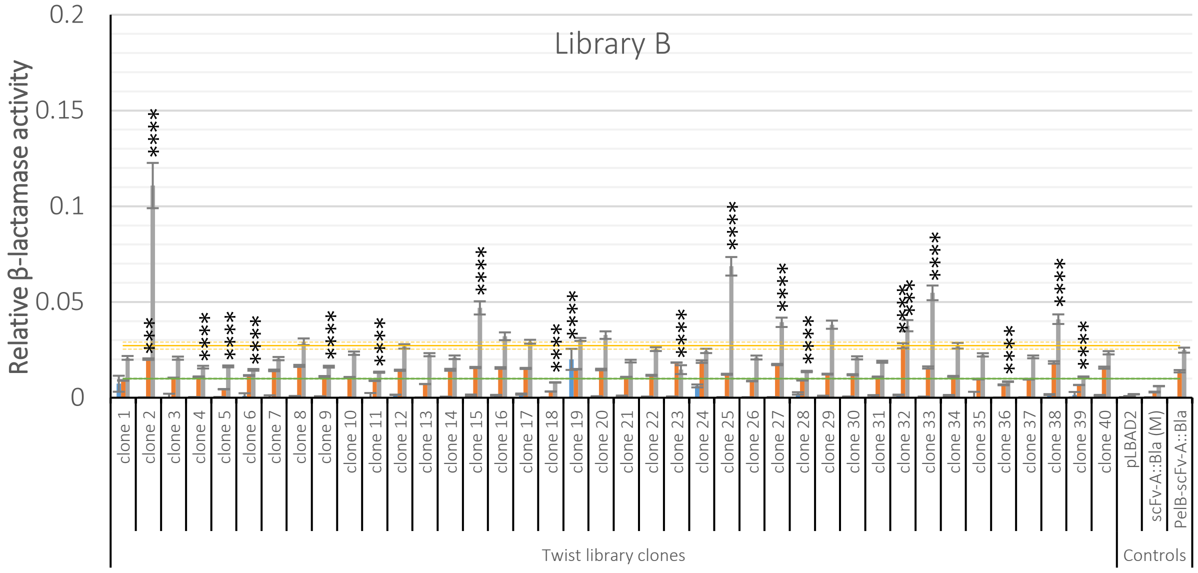
Library B
Relative β-lactamase activity
Twist library clones
Controls

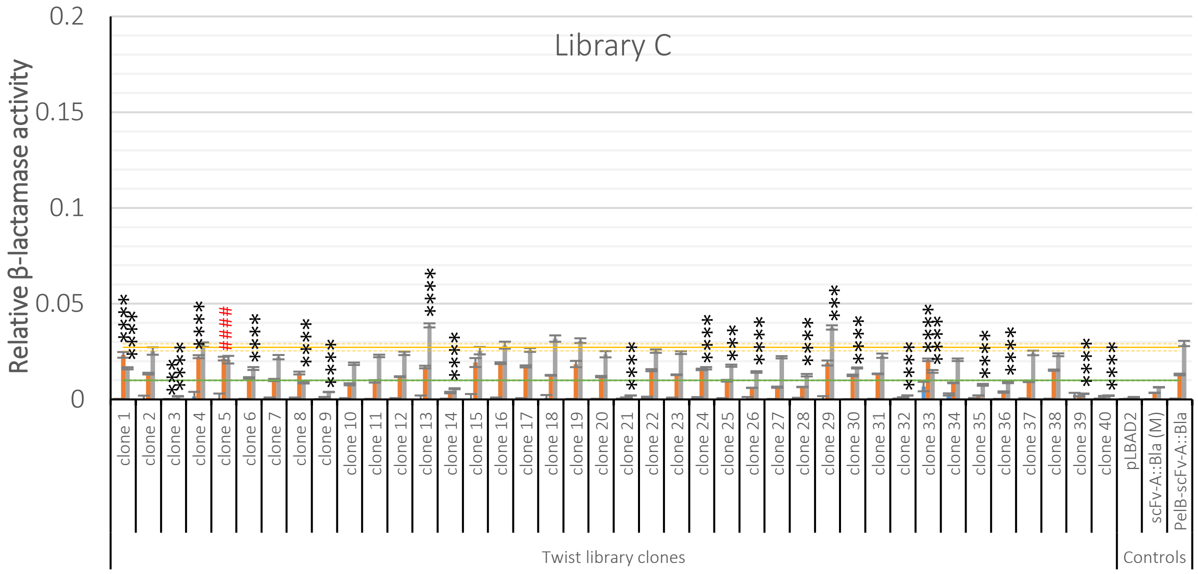
Library C
Relative β-lactamase activity
Twist library clones
Controls

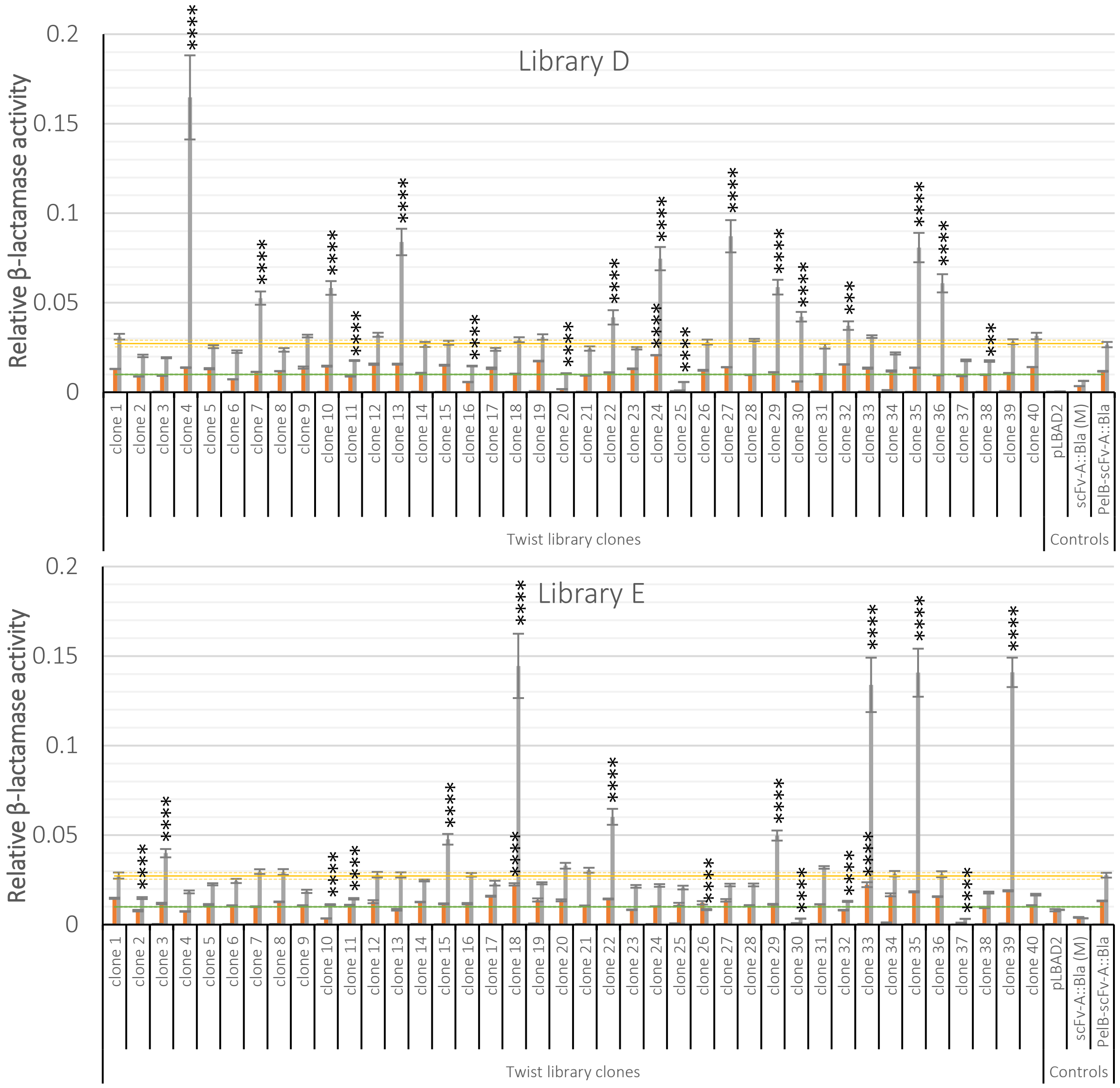

Supplemental Figure S6. β-lactamase activity screen of chemically synthesised signal peptide library. *E. coli* BL21-A transformed with pSCREEN clones containing the CS library signal peptides fused to scFv::*bla* were grown at 25 °C and induced with 0.02% arabinose after 2 hours growth. β-lactamase activity of cultures was measured by hydrolysis of nitrocefin before (2 h) and after induction (8 h and 24 h). The average β-lactamase activity of PelB^{sp} at 8 and 24 hours is shown by the green and yellow horizontal lines respectively. Error bars represent the 5% uncertainty in the calculation of the slopes corresponding to β-lactamase activity (n=2), which is expressed in terms of change in OD_{495} per minute per OD_{600}. Asterices indicate values statistically significant (**** $p < 0.0001$; *** $0.001 > p > 0.0001$) compared to the control (PelB^{sp}scFv::*bla*) after selecting adjusted $p < 0.001$ as the level of significance during statistical analysis of data. #Indicates data excluded from the statistical analysis as failed the normality test after selecting $p<0.05$ as the level of significance (n=200).

a.

b.

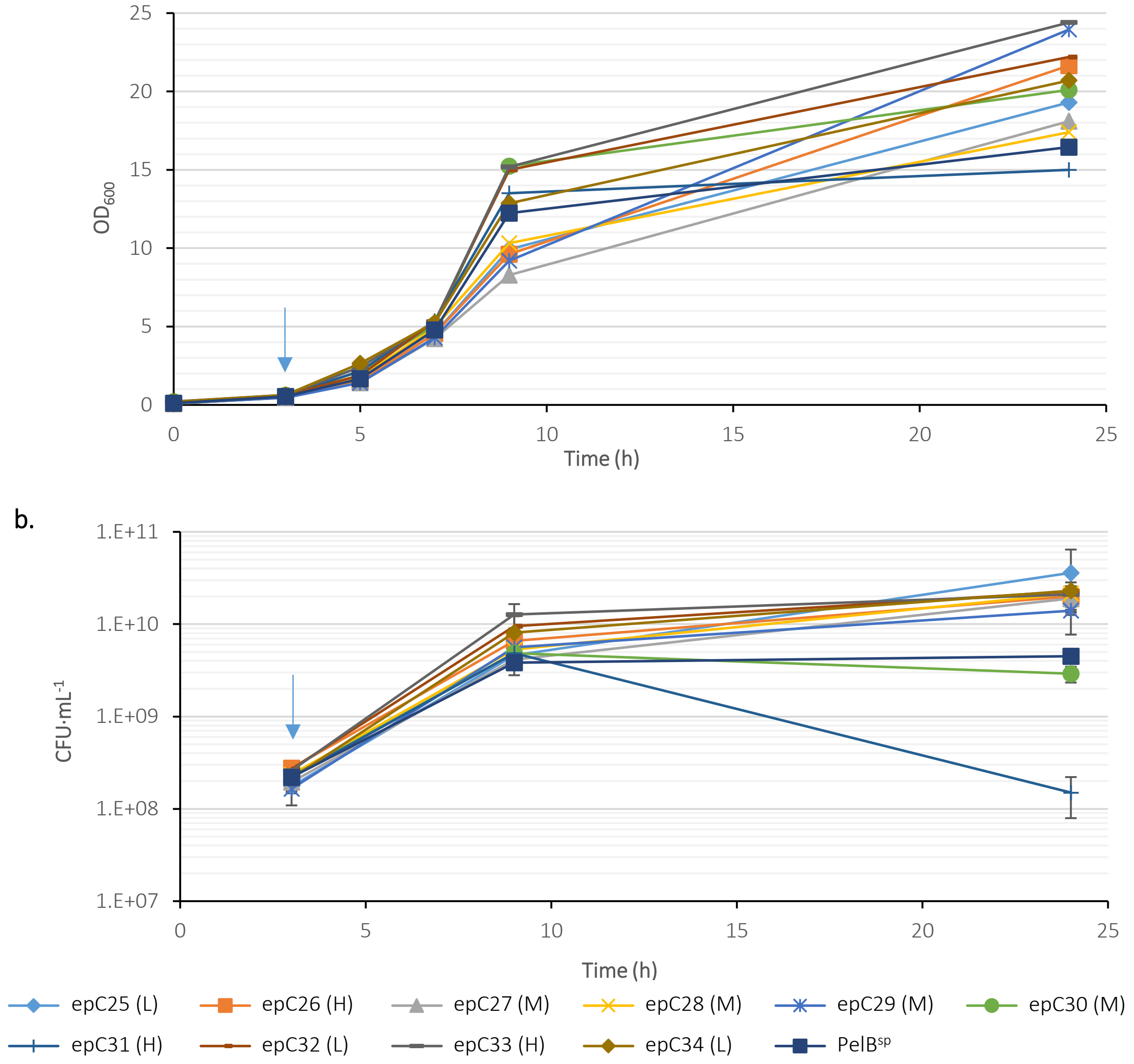

Supplemental Figure S7. Growth and cell viability of clones from the epPCR library. *E. coli* BL21-A carrying vectors coding for different signal peptides from the epPCR library (epC26-epC34) or PelB^{sp} fused to scFv::Bla were grown at 25 °C and induced with 0.02% arabinose at an OD_{600} ≈ 0.5 (blue arrow). The OD_{600} **(a)** and CFU **(b)** of the culture were measured at intervals post-inoculation. Clones are labelled as high (H), medium (M) and low (L) depending on their β-lactamase activity. Data shown are mean values of two replica flasks and error bars are ±1 standard deviation.

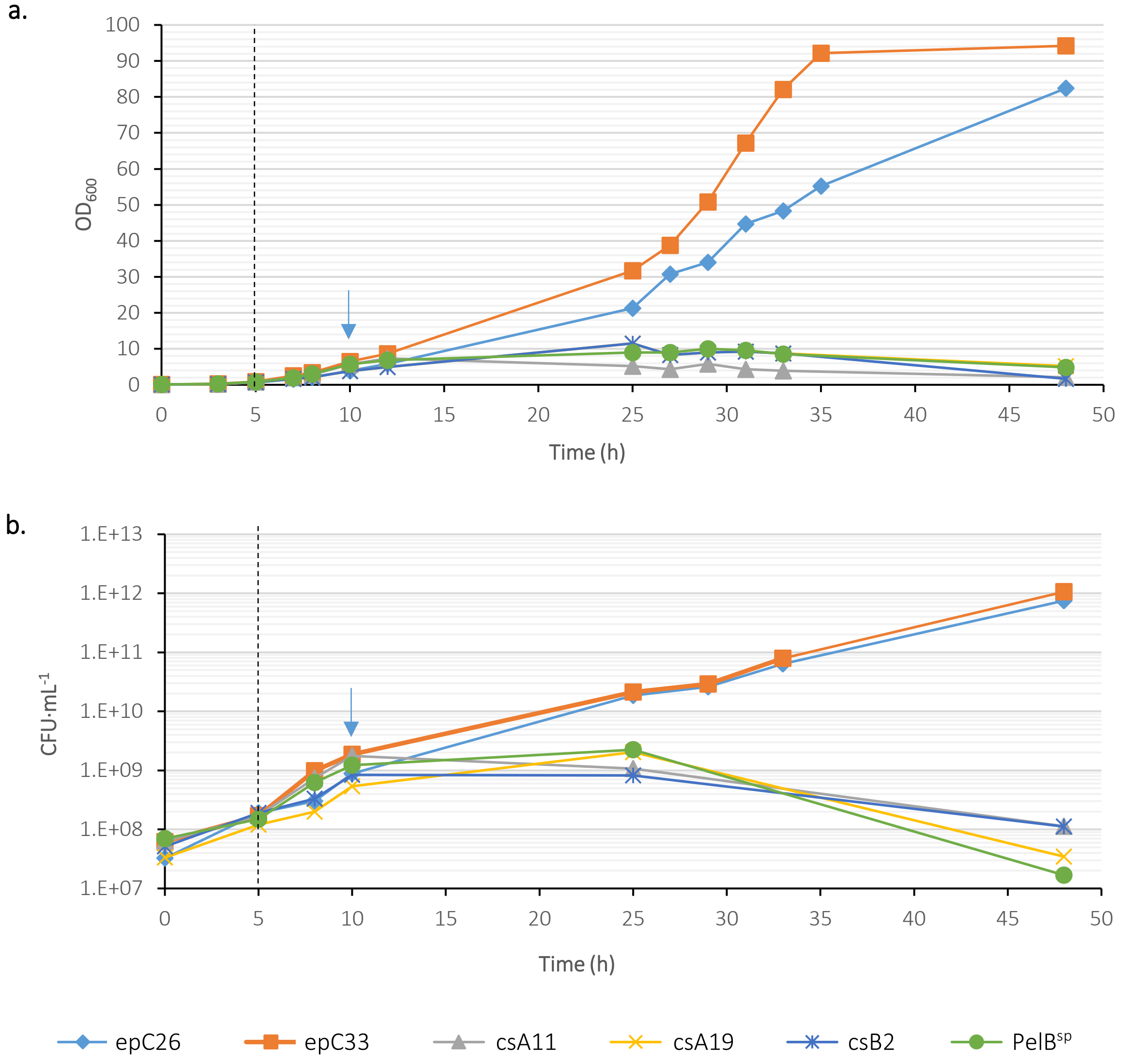

Supplemental Figure 8. Fed-batch fermentation for the production of scFv::Bla induced at low cell density. *E. coli* BL21-A carrying vectors coding for different signal peptides fused to scFv::Bla were grown in the Ambr® 250 modular fermentation system at 25 °C and induced with 0.02% arabinose at an OD_{600} of 0.5 (dotted line). The feed was started at 10 h (blue arrow). The OD_{600} **(a)** and CFU **(b)** of the culture were measured at intervals post-inoculation.

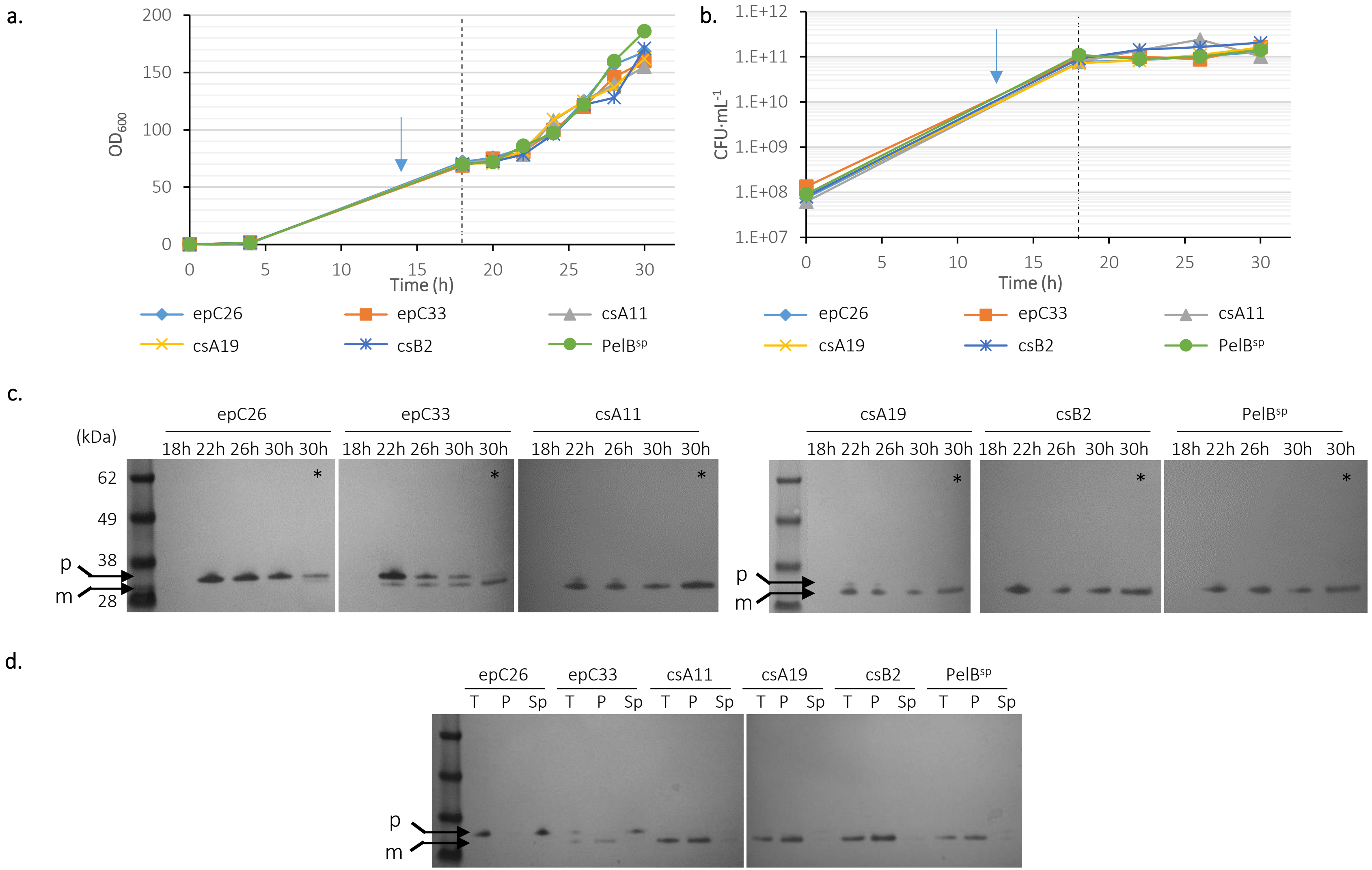
a.
200
150
100
50
0
OD600
0 5 10 15 20 25 30
Time (h)
epC26
epC33
csA11
csA19
csB2
PelBsp
b.
1.E+12
1.E+11
1.E+10
1.E+09
1.E+08
1.E+07
CFU·mL-1
0 5 10 15 20 25 30
Time (h)
epC26
epC33
csA11
csA19
csB2
PelBsp
c.
(kDa)
62
49
38
28
p
m
epC26
epC33
csA11
csA19
csB2
PelBsp
18h 22h 26h 30h 30h
18h 22h 26h 30h 30h
18h 22h 26h 30h 30h
18h 22h 26h 30h 30h
18h 22h 26h 30h 30h
18h 22h 26h 30h 30h
*
d.
epC26
epC33
csA11
csA19
csB2
PelBsp
T P Sp
p
m

Supplemental Figure S9. Fed-batch fermentation for the production of scFv using 0.02% arabinose induction. *E. coli* BL21-A carrying vectors coding for different signal peptides fused to scFv were grown in the Ambr® 250 modular fermentation system at 30 °C and induced with 0.02% arabinose at an OD_{600} ≈ 70 - 80 (dotted line) whereupon the temperature was decreased to 25 °C. The feed was started after 14 hours of growth (blue arrow). The OD_{600} **(a)** and CFU **(b)** of the culture were measured at intervals post-inoculation. Western blotting was used to detect scFv in whole cell lysates and culture medium (*) samples **(c)** and in total protein (T), periplasm (P) and spheroplast (Sp) fractions **(d)**. Arrows show precursor (p) and mature (m) forms of scFv.

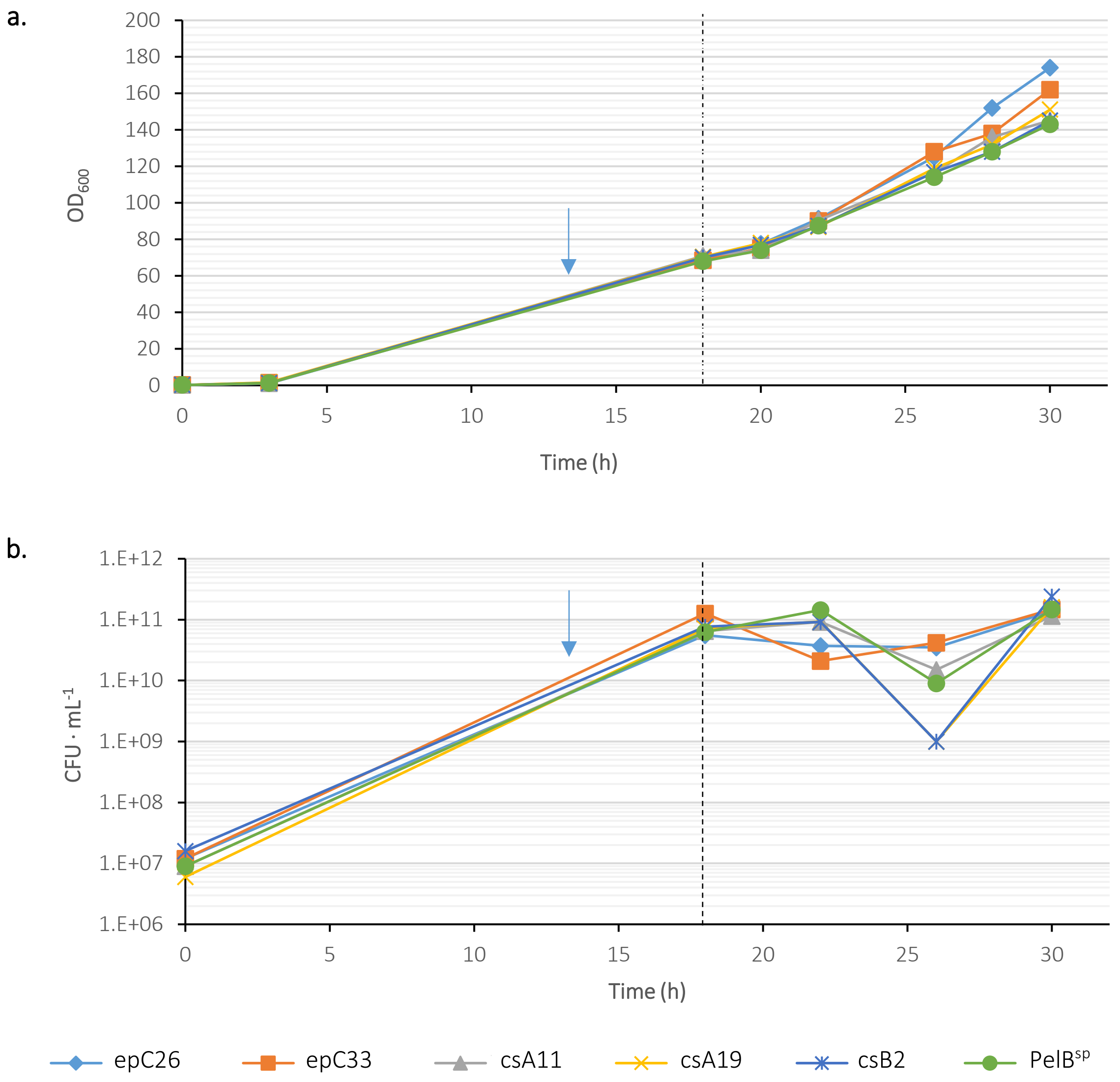

Supplemental Figure S10. Fed-batch fermentation for the production of scFv using 0.2% arabinose induction. *E. coli* BL21-A carrying vectors coding for different signal peptides fused to scFv were grown in the Ambr® 250 modular fermentation system at 30 °C and induced with 0.2% arabinose at an OD_{600} ≈ 70 - 80 (dotted line), whereupon the temperature was decreased to 25 °C. The feed was started at 14 h (blue arrow). The OD_{600} **(a)** and CFU **(b)** of the culture were measured at intervals post-inoculation.

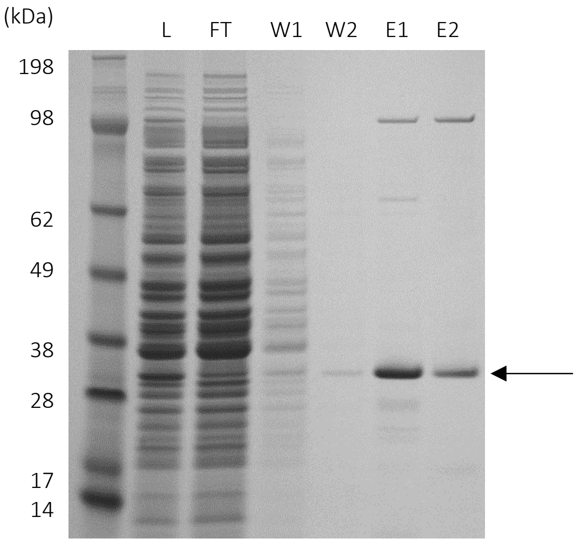

Supplemental Figure S11. Purification of scFv 13R4. SDS-PAGE gel showing the clarified culture medium loaded (L) into the HisTrap FF column, the flow-through (FT) from the column, the washing steps (W1, W2) and the eluted material (E1 and E2).

Supplemental Table S1. Plasmids used in this study.

Plasmid name	Description	Source
pLBAD2	Medium copy number, pMB1 origin, Kan^R p^{araBAD} expression vector with *Nde*I-*Sal*I cloning sites.	Cobra Biologics
pCR®-Blunt	PCR cloning vector, pUC origin, Kan^R.	Life Technologies
ptrc2-STII-scFv13R4	ptrc2 vector used as source of the sequence coding for $STII^{sp}$-scFv13R4 (Kan^R). Contains extra alanine residue at start of scFv.	Cobra Biologics
pLBAD2-STII-scFv13R4+A	pLBAD2 expressing $STII^{sp}$-scFv13R4. Contains extra alanine residue at start of scFv.	This work
pLBAD2-DsbA-scFv13R4+A	pLBAD2 expressing $DsbA^{sp}$-scFv13R4. Contains extra alanine residue at start of scFv.	This work
pLBAD2-PelB-scFv13R4+A	pLBAD2 expressing $PelB^{sp}$-scFv13R4. Contains extra alanine residue at start of scFv.	This work
pLBAD2-STII-scFv13R4	pLBAD2 expressing $STII^{sp}$-scFv13R4.	This work
pLBAD2-DsbA-scFv13R4	pLBAD2 expressing $DsbA^{sp}$-scFv13R4.	This work
pLBAD2-PelB-scFv13R4	pLBAD2 expressing $PelB^{sp}$-scFv13R4.	This work
pET163R4	pET-23d(+) vector used for cytoplasmic expression of scFv 13R4 (Amp^R). Used as source of scFv 13R4.	24
Expression of β-lactamase		
pUC18	High copy number vector used as source of *bla* gene.	45
pLBAD2-Bla-*bla*	pLBAD2 expressing β-lactamase with its own signal peptide.	This work
pLBAD2-*bla*	pLBAD2 expressing β-lactamase with no signal peptide.	This work
Expression of scFv 13R4::β-lactamase		
pMK-T-scFv13R4-linker-Bla	Source of the sequence coding for a fragment of scFv13R4, a 4 amino acid linker and the β-lactamase. Kan^R.	GeneArt
pLBAD2-STII-scFv13R4::Bla	pLBAD2 expressing $STII^{sp}$- scFv13R4::*bla*.	This work
pLBAD2-DsbA-scFv13R4::Bla	pLBAD2 expressing $DsbA^{sp}$- scFv13R4::*bla*.	This work
pLBAD2-PelB-scFv13R4::Bla	pLBAD2 expressing $PelB^{sp}$- scFv13R4::*bla*.	This work
pLBAD2-Bla-scFv13R4::Bla	pLBAD2 expressing Bla^{sp}- scFv13R4::*bla*.	This work
pLBAD2-scFv13R4::Bla	pLBAD2 expressing scFv13R4::*bla*.	This work
**Vectors used for the development of the β-lactamase screening**		
pMA-RQ-BspQIPelBBspQI	GeneArt vector (Amp^R) containing the sequence coding for $PelB^{sp}$ flanked by *Bsp*QI restriction sites, used as a template for error-prone PCR.	GeneArt
pLBAD2ΔBspQI-STII-scFv13R4::Bla	pLBAD2-STII-scFv13R4::Bla derivative in which the *Bsp*QI restriction site within the plasmid backbone has been removed (Kan^R).	This work
pSCREEN	Signal peptide screening vector, pLBAD2ΔBspQI -STII-scFv13R4::Bla derivative where $STII^{sp}$ is replaced by *Bsp*QI*Xba*I*Bsp*QI linker using *Nde*I and *Pvu*II.	This work

Supplemental Table S2. Oligonucleotide primers used in this work.

**Primer name**	**Sequence 5' to 3'**
scFv-F	AAACAGCATATGAAAAAGAATATCGCAT
scFv-R	CTTCTCTCATCCGCCAAAAC
STII-mut-F	TACAAATGCCTATGCAGAGGTGCAGCTGGTGG
STII-mut-R	CCACCAGCTGCACCTCTGCATAGGCATTTGTA
DsbA-mut-F	AGCGCATCGGCGGAGGTGCAGCTG
DsbA-mut-R	CAGCTGCACCTCCGCCGATGCGCT
PelB-mut-F	CCGGCGATGGCCGAGGTGCAGCTG
PelB-mut-R	CAGCTGCACCTCGGCCATCGCCGG
Bla-SalI-F	GTAAACTTGGTCGTCGACTTACCAATGCTTAATCA GTGAGGCACC
Bla-NdeI-R	TGAAAAAGGAAGCATATGAGTATTCAACATTTCCG TGTCGCCCTT
Cyt-Bla-F	TGCCTTCCTGTTCATATGCACCCAGAAACGCTG
LBADtrc R	ATCAGACCGCTTCTGCGTTC
NdeI-F	GCTACAAATGCCCATATGGAGGTGCAGCTGGTG
Seq Bla R	GCACCCAACTGATCTTCAGC
BspQI-F	CGTCTTTTACTGGCTCTACTCGCTAACCAAACCGG
LBADilac-F	AAGGGAGAAAGGCGGACAGG
Mut-F	GCATCAACGAGCTCGCTCTTCTATG
Mut-R	GCACCCAACTGATCTTCAGC
Oligonucleotides for introduction of sequences	
DsbA-Fa	TATGAAAAAGATTTGGCTGGCGCTGGCT
DsbA-Fb	GGTTTAGTTTTAGCGTTTAGCGCATCGGCGGCCG AGGTGCAG
DsbA-Ra	CTGCACCTCGGCCGCCGATGCGCTAAAC
DsbA-Rb	GCTAAAACTAAACCAGCCAGCGCCAGCCAAATCT TTTTCA
PelB-F	TATGAAATACCTGCTGCCGACCGCTGCTGCTGGT CTGCTGCTCCTCGCTGCCCAGCCGGCGATGGCC GCCGAGGTGCAG
PelB-R	CTGCACCTCGGCGGCCATCGCCGGCTGGGCAGC GAGGAGCAGCAGACCAGCAGCAGCGGTCGGCAG CAGGTATTTCA
Bla-F	TATGAGTATTCAACATTTCCGTGTCGCCCTTATTC CCTTTTTTGCGGCATTTTGCCTTCCTGTTTTTGCT GAGGTGCAG
Bla-R	CTGCACCTCAGCAAAAACAGGAAGGCAAAATGCC GCAAAAAAGGGAATAAGGGCGACACGGAAATGTT GAATACTCA
BspQIXbaIBsp QI-F	TATGTGAAGAGCTCTAGAGCTCTTCTGCCGAGGT GCAG
BspQIXbaIBsp QI-R	CTGCACCTCGGCAGAAGAGCTCTAGAGCTCTTCA CA

Supplementary materials and methods

Plasmid construction for scFv and bla expression

The gene encoding scFv 13R4 (24; an anti-*E. coli* β-galactosidase scFv) and an associated N-terminal STII signal peptide and C-terminal myc-His tag were amplified by PCR from ptrc2-STII-scFv13R4 using primers scFv-F and scFv-R, generating a *Nde*I-*Sal*I fragment, which was ligated into cloning vector pCR®-Blunt (Life Technologies). The STII^{sp}-scFv fragment was excised from PCR®-Blunt with *Nde*I and *Sal*I and ligated into *Nde*I-*Sal*I digested pLBAD2, resulting in pLBAD2-STII-scFv13R4+A. To replace STII^{sp} with PelB^{sp} or DsbA^{sp}, the nucleotides encoding STII^{sp} were removed by digestion with *Nde*I and *Pvu*II and replaced with chemically synthesised overlapping oligonucleotides encoding either PelB^{sp} (oligonucleotides PelB-F + PelB-R) or DsbA^{sp} (oligonucleotides DsbA-Fa, DsbA-Fb, DsbA-Ra and DsbA-Rb). Oligonucleotides were mixed, heated to 95 °C for 5 minutes then allowed to cool to room temperature. Annealed nucleotides were phosphorylated using T4 polynucleotide kinase (New England BioLabs) and ligated into *Nde*I-*Pvu*II digested, dephosphorylated pLBAD2-STII-scFv13R4+A resulting in pLBAD2-PelB-scFv13R4+A or pLBAD2-DsbA-scFv13R4+A. These three scFv-expressing constructs contained an additional alanine residue between the signal peptide and the scFv; this is because an extra alanine residue was added to scFv 13R4 when pET163R4 was constructed (24). The nucleotides encoding the extra alanine were removed by overlap extension PCR (46) resulting in plasmids pLBAD2-STII-scFv13R4, pLBAD2-DsbA-scFv13R4 and pLBAD2-PelB-scFv13R4. All experiments in this study used scFv 13R4 with this extra alanine residue deleted.

Plasmid construction for bla and scFv::bla expression

The *bla* gene and its native signal peptide were PCR amplified on an *Nde*I-*Sal*I fragment from pUC18 using primers Bla-SalI-F and Bla-NdeI-R and ligated into *Nde*I-*Sal*I digested pLBAD2, resulting in pLBAD2-Bla-*bla*. To generate pLBAD2-*bla*, expressing *bla* without a signal peptide, the *bla* gene was amplified from pLBAD2-Bla-*bla* using primers Cyt-Bla-F (introducing

an *Nde*I site and start codon) and LBAD-trcR; the PCR fragment was cloned via pCR®-Blunt, *Nde*I-*Sal*I digested and ligated into *Nde*I-*Sal*I digested pLBAD2.

Plasmids expressing scFv::*bla* fusions with $STII^{sp}$, $DsbA^{sp}$ and $PelB^{sp}$ were constructed by *Not*I-*Sal*I digestion of pLBAD2-STII-scFv13R4, pLBAD2-DsbA-scFv13R4 and pLBAD2-PelB-scFv13R4. A fragment comprising scFv13R4, a four amino acid linker and *bla* was excised from pMK-T-scFv13R4-linker-Bla using *Not*I-*Sal*I digestion and ligated into the pLBAD2 fragments generating pLBAD2-STII-scFv13R4::Bla, pLBAD2-DsbA-scFv13R4::Bla and pLBAD2-PelB-scFv13R4::Bla. A plasmid expressing Bla^{sp}-scFv::*bla* was generated from pLBAD2-STII-scFv13R4::Bla. The $STII^{sp}$ was excised with *Nde*I and *Pvu*II, and replaced with annealed phosphorylated oligonucleotides Bla-F and Bla-R as above, yielding pLBAD2-Bla-scFv13R4::Bla. A plasmid expressing scFv::*bla* with no signal peptide was generated using pLBAD2-STII--scFv13R4::Bla as a template for PCR with primers NdeI-F and Seq-Bla-R to generate a scFv::*bla* fragment, which was cloned into *Nde*I-*Sal*I digested pLBAD2 to form pLBAD2-scFv13R4::Bla.

Screening – design of constructs

The screening system utilised the type IIs restriction enzyme *Bsp*QI, which cleaves DNA away from its sequence recognition site and thereby leaves a 3-nucleotide overhang on the 5' strand (Supplemental Fig. S3). These overhangs do not form part of the DNA sequence recognised by the restriction enzyme, so the enzyme can be used to cut DNA without introducing mutations into the resultant ligated plasmid DNA sequence. Accordingly, the screening plasmid pSCREEN contained a cloning site with *Bsp*QI recognition sites located so that they would be excised from the linearised plasmid, leaving overhangs complementary to the ATG start codon of the signal peptide and the final GCC codon of $PelB^{sp}$. The signal peptide library was generated on a fragment containing *Bsp*QI recognition sites outside the signal peptide sequence, likewise allowing cleavage without introduction of mutations. An additional advantage of using this approach for a mutagenic screen is that the complementary overhangs

required for ligation corresponded to two residues of the resultant signal peptide that were invariant: the start ATG codon; and the GGC codon encoding the last amino acid of the signal peptide recognised by signal peptidase, both of which are required for functionality.

Plasmid construction for screen

Screening plasmid pSCREEN was constructed in two stages from pLBAD2-STII-scFv13R4::Bla (Supplemental Fig. S3). First, a *Bsp*QI restriction site present in the *araBAD* promoter region of pLBAD2-STII-scFv13R4::Bla was removed via three rounds of PCR using Phusion DNA polymerase (New England Biolabs). First, the p^{araBAD}-$STII^{sp}$ region of pLBAD2-STII-scFv13R4::Bla was amplified using primers BspQI-F and STII-mut-R, the former eliminating the *Bsp*QI site. The product of the first round PCR was then used as a reverse primer in a second round PCR using LBADilac-F as a forward primer and pLBAD2-STII-scFv13R4::Bla as a template, generating an *Bsp*EI-*ori*-*araC*-p^{araBAD}-$STII^{sp}$-*Nde*I fragment. The resultant fragment was then amplified in a third round of PCR with primers LBADilac-F and STII-mut-R. The *Bsp*EI-*ori*-*araC*-p^{araBAD}-$STII^{sp}$-*Nde*I fragment was digested with *Bsp*EI and *Nde*I and ligated into *Bsp*EI-*Nde*I digested pLBAD2-STII-scFv13R4::Bla, generating pLBAD2ΔBspQI-STII-scFv13R4::Bla. Next, $STII^{sp}$ was excised with *Nde*I and *Pvu*II and replaced with annealed oligonucleotides containing a *Bsp*QI-*Xba*I-*Bsp*QI linker region to generate pSCREEN.

Signal peptide screen

The PelB signal peptide sequence flanked by *Bsp*QI sites was synthesised by GeneArt (Thermo Fisher Scientific) and provided on a plasmid (pMA-RQ-BspQIPelBBspQI), which was linearized using *Sfi*I and used as a template for epPCR using the GeneMorphII random mutagenesis kit (Agilent, Stockport, UK) and primers Mut-F and Mut-R. The PCR product obtained from this first round epPCR reaction was used as template for a second round reaction, and so on until four successive rounds of epPCR were completed (epPCR1-4). A fifth pool, epPCR5, comprised a mixture of pool epPCR1-4. Independently, a chemically

synthesised signal peptide library was generated by Twist Biosciences (San Francisco, USA) comprising 10 000 nucleotides containing 2-4 mutations each. This CS oligonucleotide library was made double stranded using PCR. The screening plasmid pSCREEN, the epPCR pools and the CS library were digested with *Bsp*QI and the inserts ligated into the vector. The ligation mixtures were electroporated into *E. coli* ElectroSHOX, recovered for 1 hour in 950 µL of LB, then transferred to 200 mL of LB plus 50 µg·mL^{-1} kanamycin in a 1 L shake flask and grown at 37 °C for 12-18 h, after which cells were harvested and plasmid DNA extracted using a PureYield® Plasmid Maxiprep Kit (Promega, Southampton, UK). Plasmid DNA was transformed into *E. coli* BL21-A and grown overnight at 37 °C in 50 mL LB plus 50 µg·mL^{-1} kanamycin. These cultures were used to make research cell banks (glycerol was added to a concentration of 20% and the mixture was stored at -80 °C) and were also used to inoculate 10 mL of LB plus 50 µg·mL^{-1} kanamycin, which was grown at 37 °C to an OD_{600} of 1-2. These cultures were serially diluted in PBS to 10^4 CFU·mL^{-1} and plated onto M-H agar containing 0.2% arabinose, 50 µg·mL^{-1} kanamycin and 3-1600 µg·mL^{-1} ampicillin before being incubated at 37 °C for 12-18 h. Colonies were selected for further analysis.

SDS-PAGE and Western blotting

4-12% Bis-Tris NuPAGE SDS-PAGE gels (Life Technologies) were generally used to evaluate the production of recombinant proteins. Seven microliters of protein sample were mixed with 2 µL of 4x NuPAGE LDS sample buffer (Life Technologies) and 1 µL of 10x NuPAGE sample reducing agent (Life Technologies), and heated for 10 min at 70 °C.

For 18% Tris-Glycine SDS-PAGE gels, culture samples were prepared by mixing 4 µL of protein sample with 5 µL 2x Tris-Glycine sample buffer (Life Technologies) and 1 µL 10x NuPAGE reducing agent. Samples were heated for 2 min at 85 °C.

1x electrophoresis running buffer was prepared by diluting 20x NuPAGE MES SDS running buffer (Life Technologies) in deionised water for 4-12% Bis-Tris NuPAGE SDS-PAGE gels or by diluting 10x Tris-Glycine SDS running buffer (Life Technologies) for 18% Tris-Glycine SDS-

PAGE gels. For reducing protein electrophoresis, 0.5 mL of NuPAGE antioxidant (Life Technologies) was added to 200 mL of running buffer and used to fill the inner chamber of the electrophoresis tank. 4-12% Bis-Tris NuPAGE gels were run for at least 45 minutes at 200 V and 18% Tris-Glycine SDS-PAGE gels were run for 3 hours at 125 V, according to the manufacturers' protocol. SeeBlue® Plus2 Pre-Stained Protein Standard (Life Technologies) was used as a molecular size marker. SDS-PAGE gels were stained using Colloidal Blue Staining (Life Technologies). SDS-PAGE gels were submerged in the fixing solution, (40 % (v/v) methanol, 10 % (v/v) glacial acetic acid) for 10 minutes, then staining solution A (20 % (v/v) methanol and 20 % (v/v) staining solution A) for 10 minutes, then staining solution B was added to a final concentration of 5 % (v/v). SDS-PAGE gels were stained for a minimum of 3 hours and de-stained with deionised water for at least 12 hours.

Proteins were transferred to a 0.45 µm nitrocellulose membrane (Life Technologies) using an Xcell II blot module (Life Technologies) at 25 V for 1 h. Transfer buffer was prepared by the addition of 20x NuPAGE transfer buffer (Life Technologies), 10 % (v/v) methanol and 1 $mL{\cdot}L^{-1}$ NuPAGE antioxidant. Membranes were blocked using 5 % (w/v) skimmed milk powder (Sigma-Aldrich) in PBS for 1 hour. Primary antibodies used were anti-His (R930-25, Life Technologies) and anti-myc (R950-25, Life Technologies) both used at 1:5 000 dilution and anti-Bla (sc-66062, Santa Cruz Biotechnology) used at 1:500 dilution, both incubated in blocking buffer for > 1 h. Membranes were washed with PBST (PBS plus 0.05 % Tween-20), incubated with 2° antibody (anti-mouse IgG conjugated to horseradish peroxidase, A4416, Sigma-Aldrich, 1:5 000 dilution) for 1 hour in blocking buffer. After washing (3 × 10 min in PBST) blots were developed using 3,3',5,5'-tetramethylbenzidine (TMB) liquid substrate (Sigma-Aldrich).

ELISA

MaxiSorp flat-bottom 96-well plates (Nunc) were coated overnight at 4 °C with 10 $µg{\cdot}mL^{-1}$ β-galactosidase (Sigma-Aldrich). The β-galactosidase solution was discarded and the plates

blocked with 200 µL of blocking buffer (5 % (w/v) milk powder in PBS) for 2 h, followed by three washes with PBS.

Total cell lysate samples were obtained by resuspending cell pellets (equivalent to 100 OD_{600}·mL) in 20 mL of 100 mM Tris-HCl pH 8 and incubated on ice. Samples were sonicated (Model 505 Sonic Dismembrator, Fisher Scientific) with 60% amplitude, for eight cycles (30 seconds on, 30 seconds off).

Periplasmic and spheroplast protein samples were obtained by modified cold osmotic shock from culture samples. Cell pellets (equivalent to 100 OD_{600}·mL) were suspended in 5 mL of ice-cold spheroplast buffer (100 mM Tris-HCl pH 8.2, 500 mM sucrose, 5 mM EDTA), then lysozyme was added to 0.8 mg·mL^{-1} followed by 5 mL of ice-cold deionised water, and incubated on ice for 5 min. $MgSO_4$ was added to a concentration of 20 mM then the samples were centrifuged (3 800 *g*, 5 min). The supernatant comprised the periplasmic protein fraction, the pellet (resuspended in 10 mL of 100 mM Tris-HCl pH 8) comprised the spheroplast fraction. Spheroplast samples were sonicated (Model 505 Sonic Dismembrator, Fisher Scientific) with 60% amplitude, for eight cycles (30 seconds on, 30 seconds off).

Cell extracts including total cell lysates, periplasmic and spheroplast protein fractions and culture medium samples were added to the plates (100 µL per well). The purified scFv13R4, used as standard reference material (see below), was also added to the plates. Samples were diluted across the plate with blocking buffer and incubated for 1 hour at room temperature. Plates were washed three times with PBS and the 1° antibody (HRP-conjugated anti-myc, R951-25, Life Technologies) was added (1:5 000 dilution in blocking buffer) and incubated at room temperature for 1 h. Plates were washed three times with PBS. TMB liquid substrate for ELISA (Sigma-Aldrich; 100 µL per well) was added; after 10 minutes, 100 µL of 1 M H_2SO_4 was added to stop the reaction. The OD_{450} of each well was measured. GraphPad was used to calculate scFv concentrations using a standard curve constructed from purified 13R4 scFv.

Calculations

Specific productivity values were calculated from densitometry and ELISA activity data, assuming that an OD_{600} of 1 is equal to 0.4 $g \cdot L^{-1}$ dry cell weight and 60% of dry cell weight is protein (47). Specific growth rate μ was calculated according to equation (2):

$$\mu = \frac{Ln\,(X_2) - Ln\,(X_1)}{t_2 - t_1} \qquad (2)$$

Where: X_1 and X_2 are biomass concentrations at times t_1 and t_2.

Statistical analysis

All the experimental data obtained from the β-lactamase activity assay was analysed using GraphPad Prism 7 software. The three datasets contained β-lactamase activity of each clone from the epPCR library (n=200), CS library (n=200) and $PelB^{sp}$ (n=40). Normal distribution was evaluated by Shapiro-Wilk normality test selecting a p-value <0.05 for the β-lactamase activity data obtained per clone (n=3); non-normally distributed data was excluded from any further analysis. The data analysis was conducted by two-way ANOVA selecting a p-value <0.05 followed by multiple comparisons using Dunnett's test for adjusted p-value of <0.001. Data obtained from each clone was compared to the control ($PelB^{sp}$-scFv::*Bla*) using Dunnett's test and values statistically significant with a p value of <0.001 are indicated.

ADDITIONAL REFERENCES

45. Norrander, J., Kempe, T. & Messing, J. Construction of improved M13 vectors using oligodeoxynucleotide-directed mutagenesis. *Gene.* **26**, 101-106 (1983).

46. Wei, H., Hu, J., Wang, L., Xu, F. & Wang, S. Rapid gene splicing and multi-sited mutagenesis by one-step overlap extension polymerase chain reaction. *Anal. Biochem.* **429**, 76-8 (2012).

47. Valgepea, K., Adamberg, K., Seiman, A. & Vilu, R. *Escherichia coli* achieves faster growth by increasing catalytic and translation rates of proteins. Mol. BioSyst. **9,** 2344-2358. (2013)

SUPPLEMENTAL FIGURES S12 – S17

Uncropped versions of SDS-PAGE gels and Western blots as shown in the paper figures. Please refer to the corresponding figure in the paper for legend.

SUPPLEMENTAL FIGURE S12 – Uncropped version of Figure 1c & d

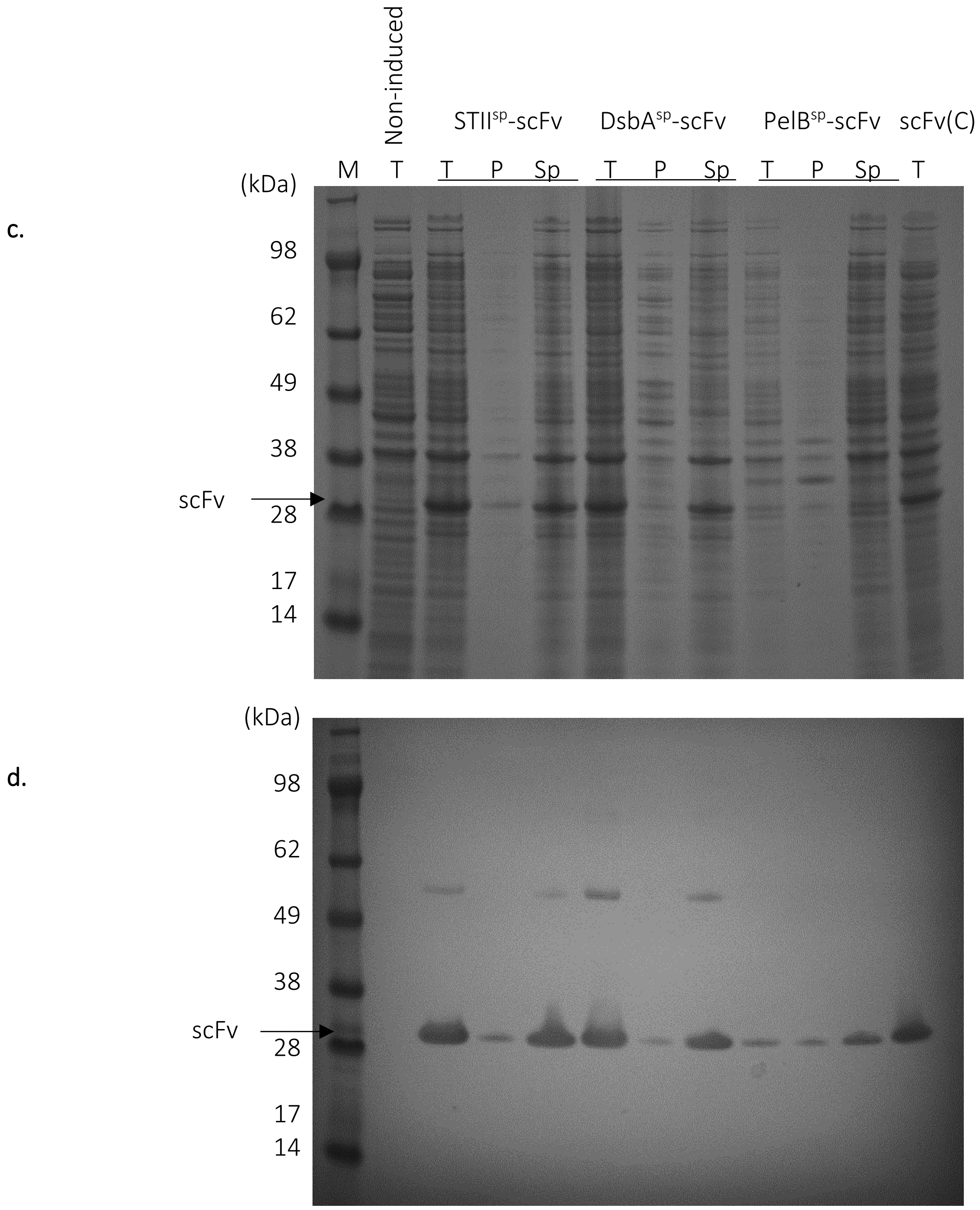

SUPPLEMENTAL FIGURE S13 – Uncropped version of Fig. 2d & e

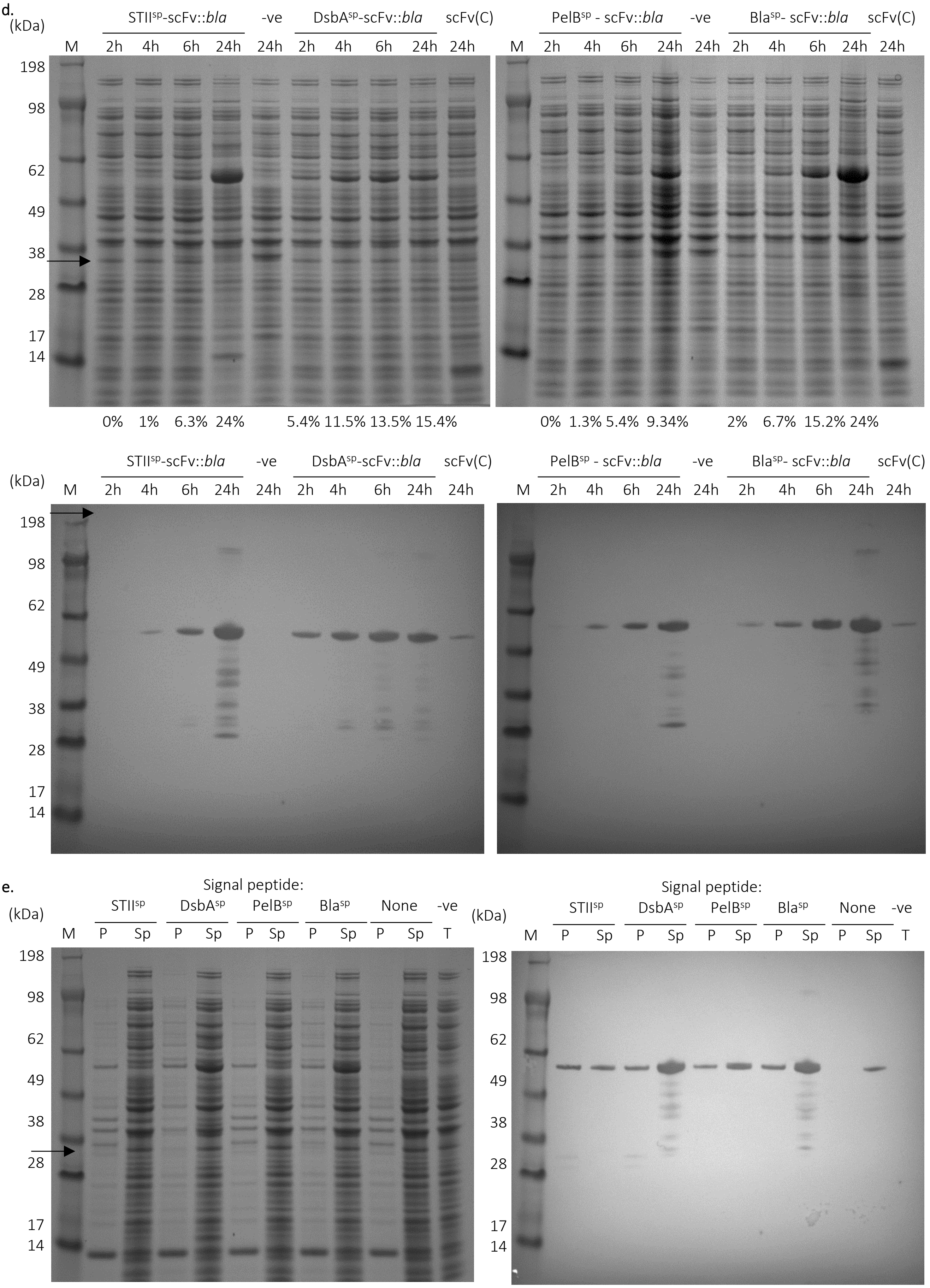

SUPPLEMENTAL FIGURE S14 – Uncropped version of Fig. 4a & b

a.

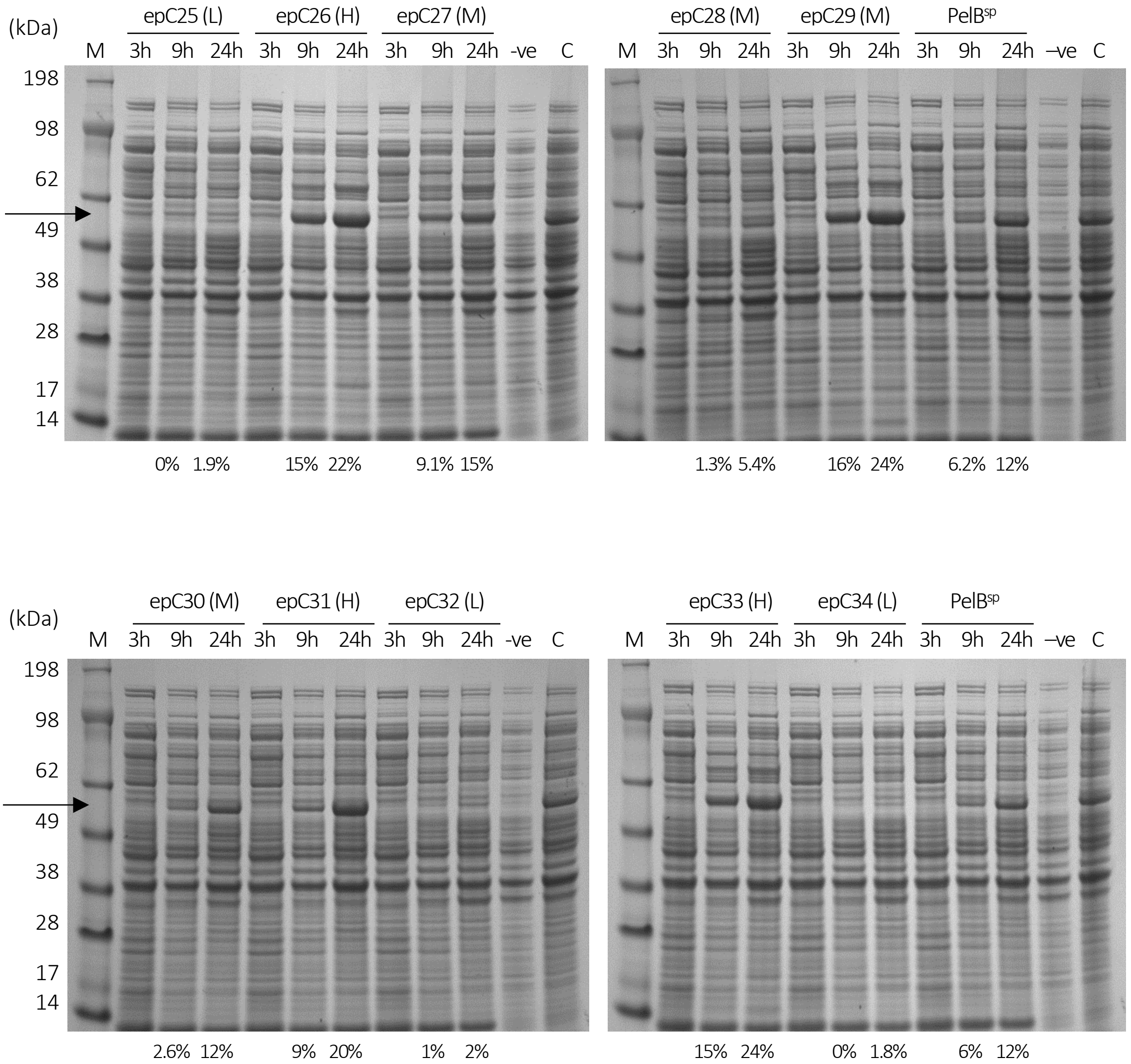

b.

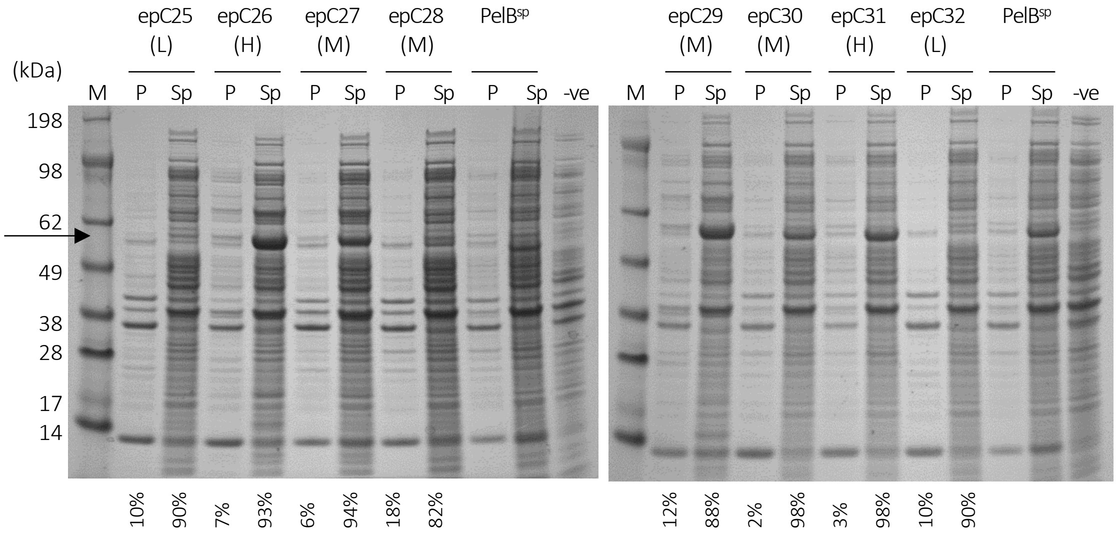

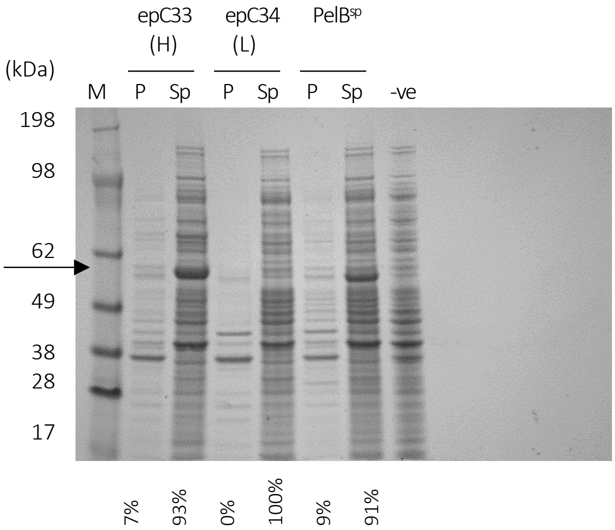
epC33
(H)
epC34
(L)
PelBsp
(kDa)
M
P
Sp
P
Sp
P
Sp
-ve
198
98
62
49
38
28
17
7%
93%
0%
100%
9%
91%

SUPPLEMENTAL FIGURE S15 – Uncropped version of Fig. 5c & d

c.

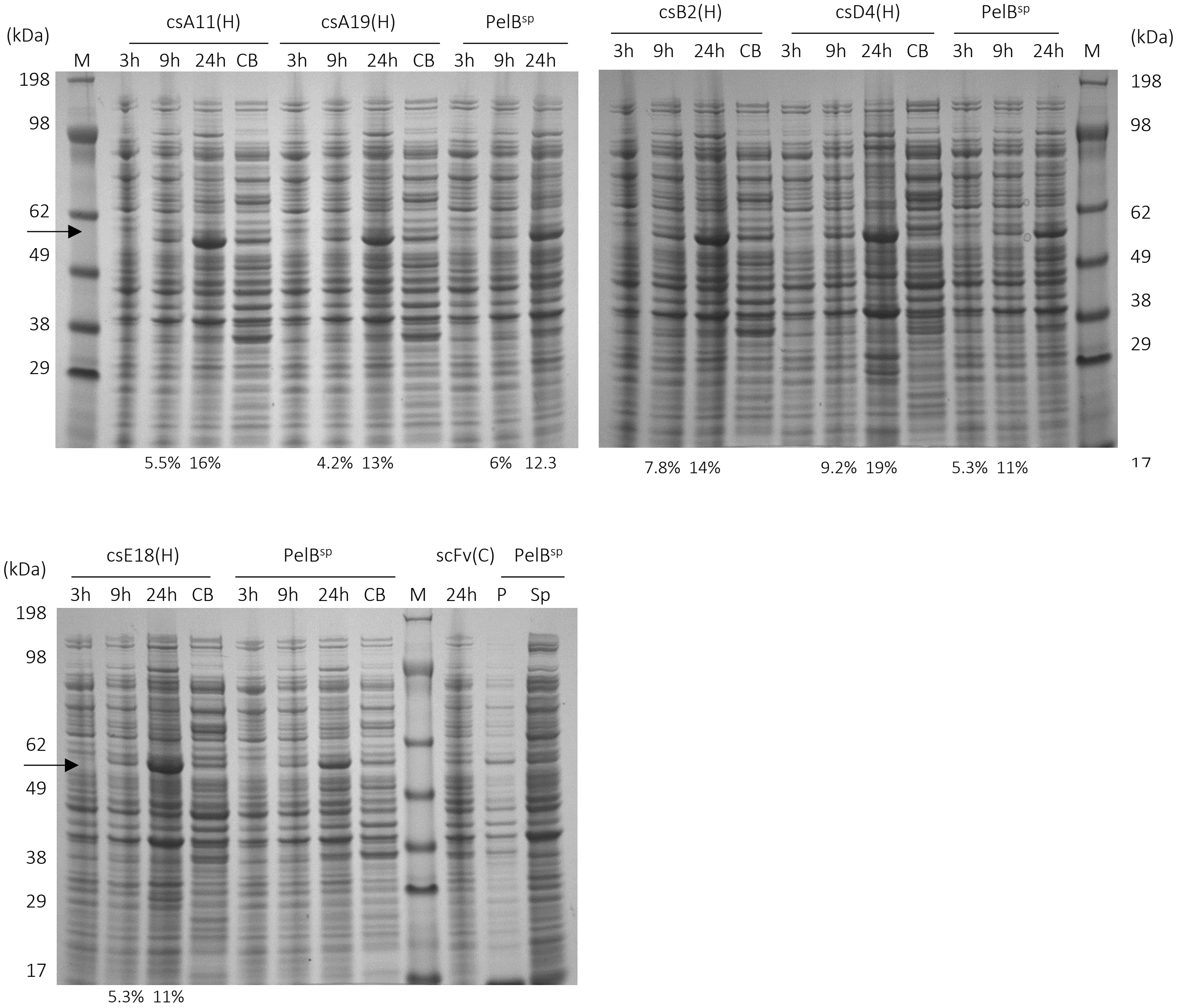

d.

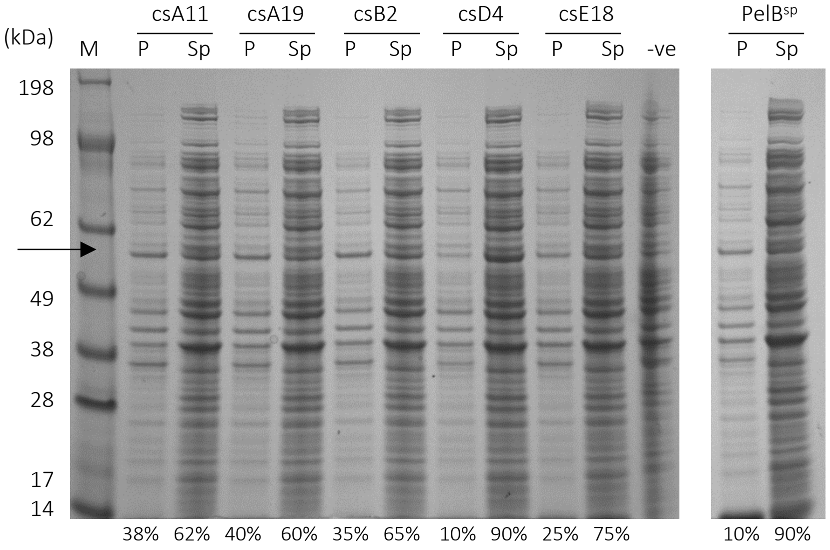

SUPPLEMENTAL FIGURE S16 – Uncropped version of Fig. 7c & d.

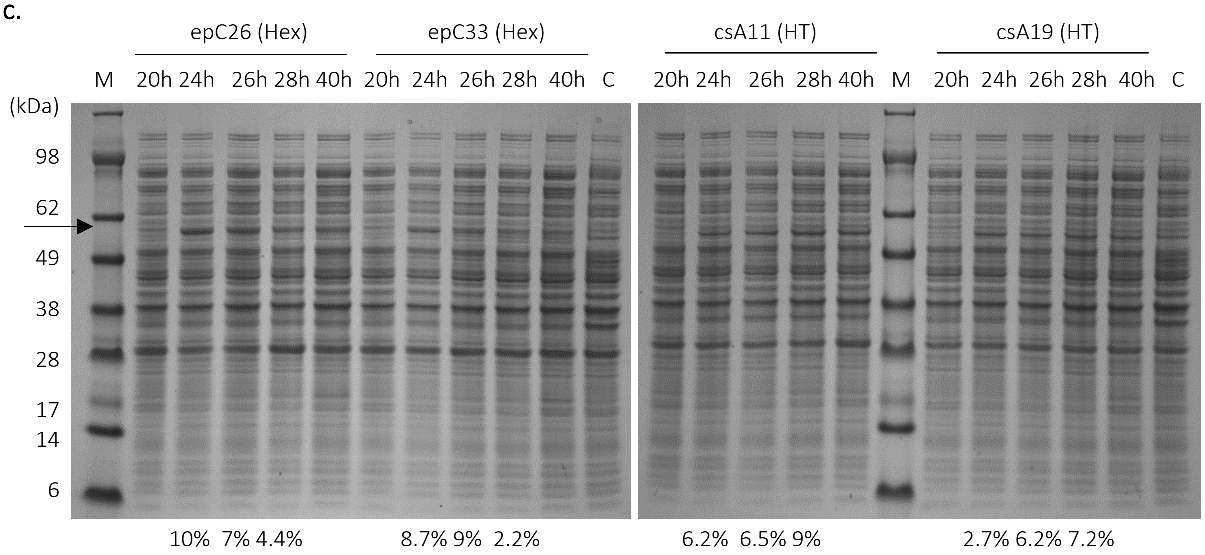

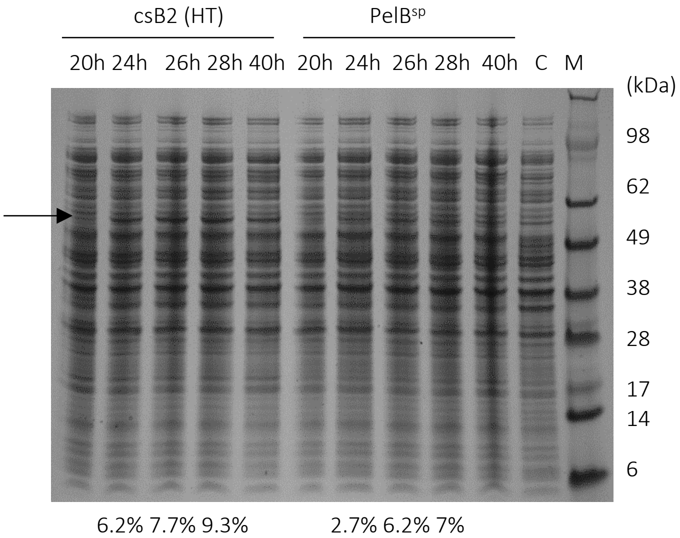

d.

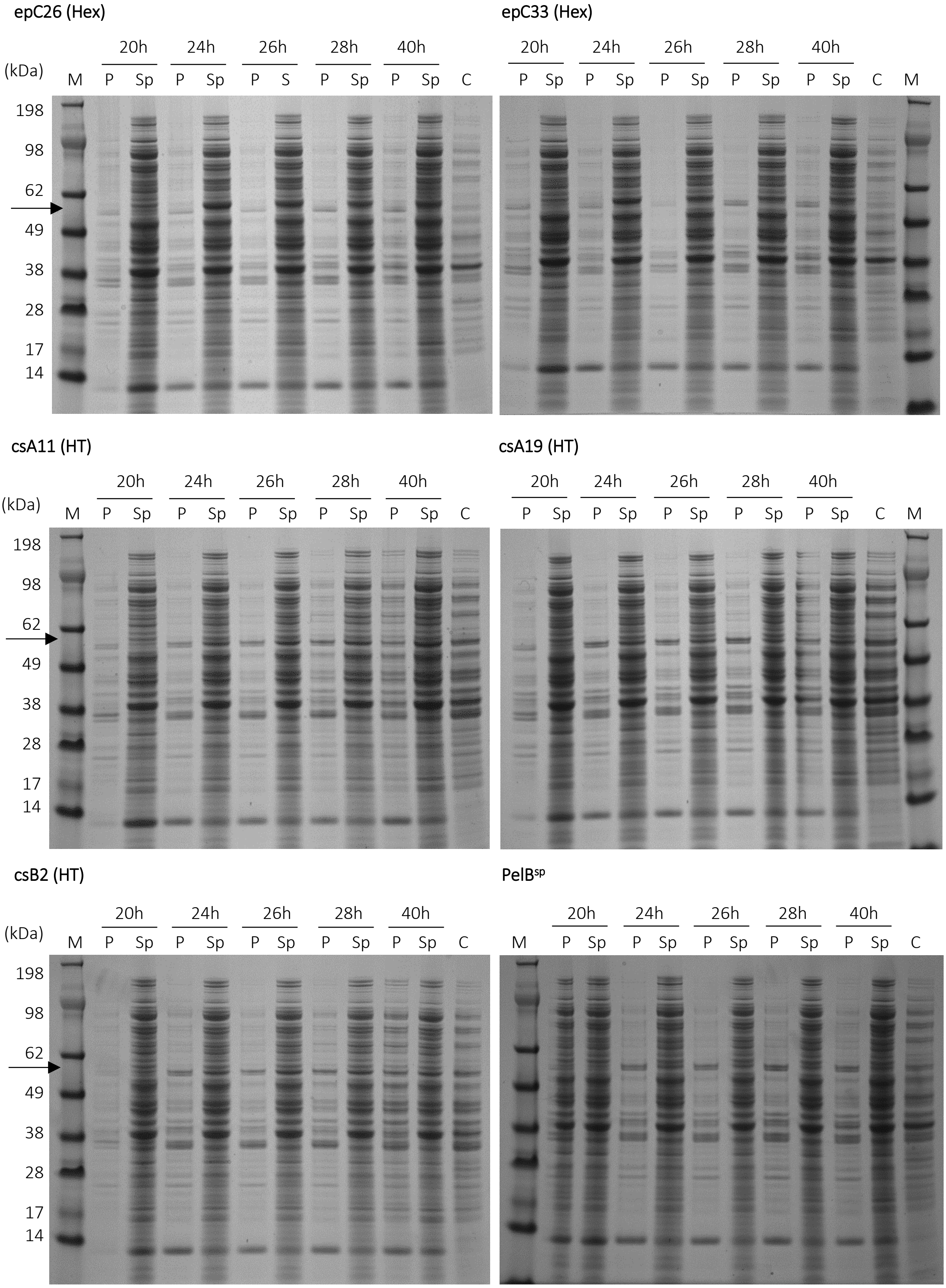
epC26 (Hex)
20h 24h 26h 28h 40h
(kDa) M P Sp P Sp P S P Sp P Sp C
198
98
62
49
38
28
17
14
epC33 (Hex)
20h 24h 26h 28h 40h
P Sp P Sp P Sp P Sp P Sp C M
csA11 (HT)
20h 24h 26h 28h 40h
(kDa) M P Sp P Sp P Sp P Sp P Sp C
198
98
62
49
38
28
17
14
csA19 (HT)
20h 24h 26h 28h 40h
P Sp P Sp P Sp P Sp P Sp C M
csB2 (HT)
20h 24h 26h 28h 40h
(kDa) M P Sp P Sp P Sp P Sp P Sp C
198
98
62
49
38
28
17
14
PelBsp
20h 24h 26h 28h 40h
M P Sp P Sp P Sp P Sp P Sp C

SUPPLEMENTAL FIGURE S17 – Uncropped version of Fig. 8a-c

a.

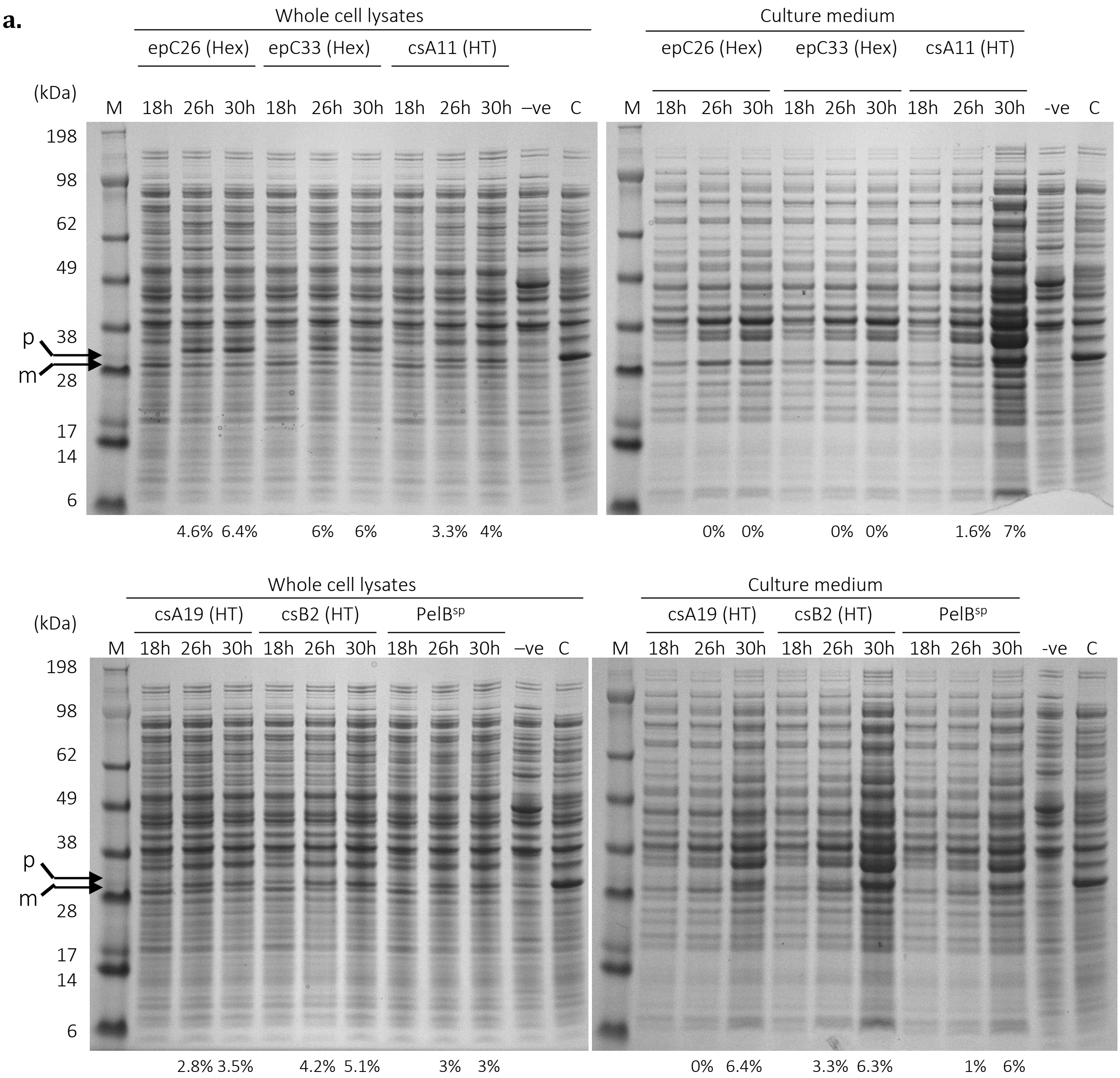

b.

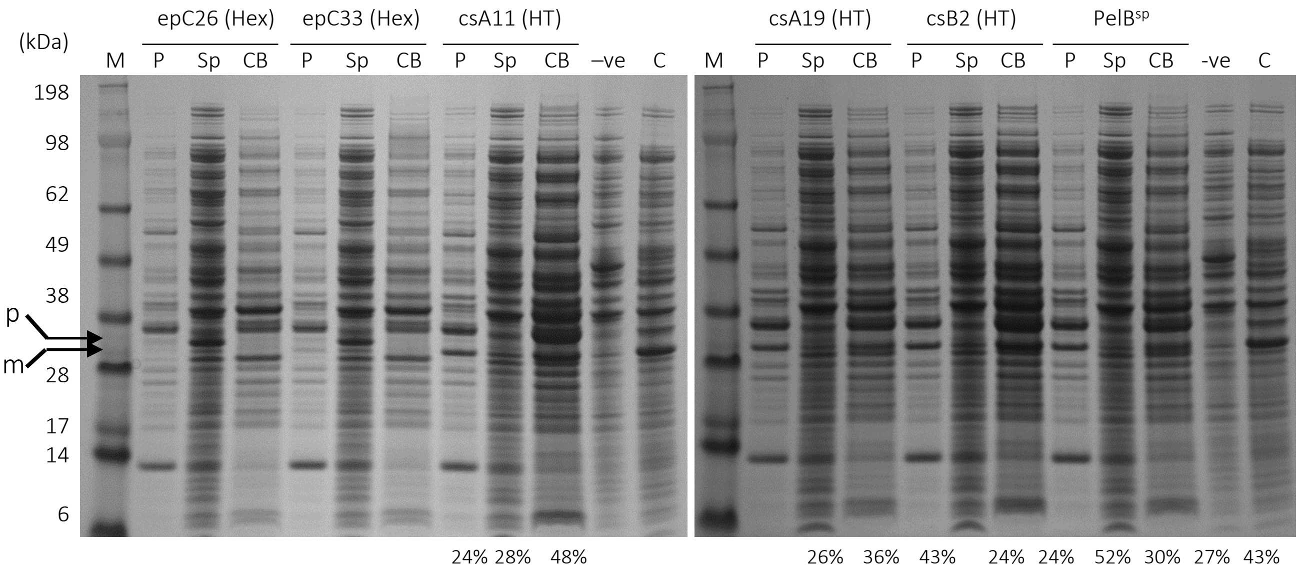
epC26 (Hex)
epC33 (Hex)
csA11 (HT)
csA19 (HT)
csB2 (HT)
PelB^sp
(kDa)
M P Sp CB P Sp CB P Sp CB –ve C
M P Sp CB P Sp CB P Sp CB -ve C
198
98
62
49
38
28
17
14
6
p
m
24% 28% 48%
26% 36% 43% 24% 24% 52% 30% 27% 43%

c.

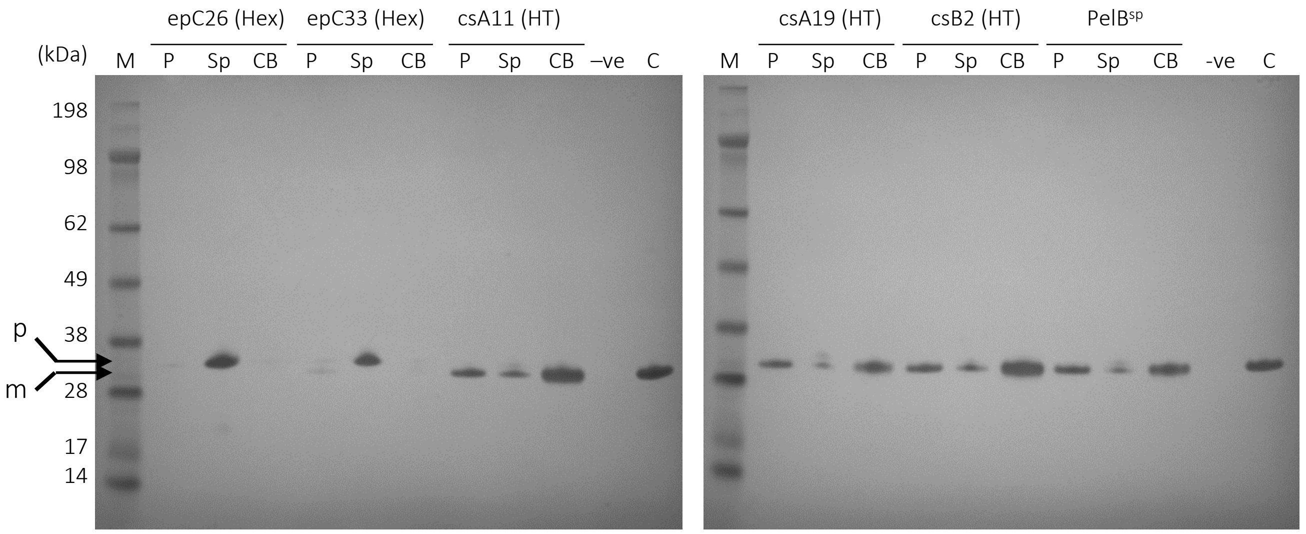
epC26 (Hex)
epC33 (Hex)
csA11 (HT)
csA19 (HT)
csB2 (HT)
PelB^sp
(kDa)
M P Sp CB P Sp CB P Sp CB –ve C
M P Sp CB P Sp CB P Sp CB -ve C
198
98
62
49
38
28
17
14
p
m
